# Precision nanomedicine for lung metastatic osteosarcoma: challenges, therapeutic strategies, and perspectives

**DOI:** 10.1016/j.mtbio.2026.103229

**Published:** 2026-05-12

**Authors:** Haozheng Li, Jing Luo, Qianli Wang, Yang Zhang, Qiujiang Li, Xiaoyong Wang, Gang Liu, Changrong Shi, Wei Zhang

**Affiliations:** a Department of Orthopedics, Sichuan Provincial People's Hospital & Sichuan Academy of Medical Sciences & Affiliated Hospital of University of Electronic Science and Technology, Chengdu, 610072, China; b State Key Laboratory of Vaccines for Infectious Diseases, Xiang an Biomedicine Laboratory, National Innovation Platform for Industry-Education Integration in Vaccine Research, Fujian Engineering Research Center of Molecular Theranostic Technology, Center for Molecular Imaging and Translational Medicine, School of Public Health, Xiamen University, Xiamen, 361102, China; c Department of Urology, Brigham and Women's Hospital, Harvard Medical School, Boston, MA, 02115, USA; d Department of Diagnostic Radiology, Yong Loo Lin School of Medicine, National University of Singapore, Singapore, 117599, Singapore

**Keywords:** Lung metastatic osteosarcoma, Nanomedicine, Metastatic microenvironment, Targeted drug delivery, Tumor-nerve interaction

## Abstract

Lung metastatic osteosarcoma (LM-OS) remains the leading cause of mortality in patients with osteosarcoma and is poorly controlled by current therapeutic regimens. The limited efficacy of conventional treatments is closely related to the complex biological features of metastatic lesions, including marked tumor heterogeneity, restrictive microenvironmental conditions, immune suppression, and emerging neural involvement within the lung niche. In recent years, nanomedicine has been increasingly explored as a mean to improve drug delivery and therapeutic effectiveness in LM-OS. A range of nanocarrier strategies has been developed to enhance metastatic targeting, modulate the tumor microenvironment, and integrate multiple therapeutic functions. In this review, we summarize recent advances in nanomedicine for LM-OS using a barrier-oriented and disease-matched framework. Representative nanocarrier platforms designed to address heterogeneity, immune clearance, limited metastatic accessibility, and tumor-nerve interactions are discussed, together with the underlying biological considerations. Current translational challenges, including safety, manufacturability, and limitations of existing preclinical models, are also examined, and perspectives on future directions for nanomedicine-based treatment of LM-OS are provided. By anchoring nanocarrier design to the biological logic of LM-OS, this review aims to provide a conceptual roadmap for developing clinically translatable precision nanotherapies.

## Graphical abstract

**Figure:**
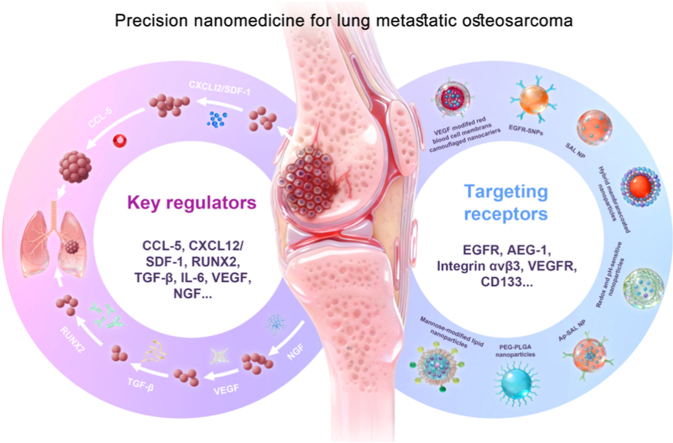

## 1 Introduction

Osteosarcoma (OS) is the most common primary malignant bone tumor in children and adolescents and is characterized by high aggressiveness and early systemic dissemination [[1], [2], [3]]. Although multimodal therapy combining neoadjuvant chemotherapy and surgical resection has improved outcomes for patients with localized disease, long-term survival has stagnated for decades [4]. This therapeutic impasse is largely driven by pulmonary metastasis, which represents the predominant cause of mortality in OS [[5], [6], [7]]. Clinically detectable lung metastases occur in approximately 20% of patients at diagnosis, while accumulating evidence suggests that occult pulmonary micrometastases are already established in most cases [4,6]. Consequently, osteosarcoma should be regarded not merely as a localized bone malignancy, but as a systemic disease with an intrinsic propensity for lung colonization.

Lung metastatic osteosarcoma (LM-OS) is biologically and therapeutically distinct from primary bone lesions. The reason lies in the genomic differences between metastatic and primary lesions, including higher mutation load and gene instability in non metastatic lesions, differential cellular mutations, and gene copy number variations. Studies have shown that MAPK signaling pathway is specifically enriched only in the stage of lung metastasis [8]. Meanwhile, lung metastases showed enhanced immunogenicity. This is manifested by higher neoantigen load, elevated PD-L1 expression level and more tumor infiltrating lymphocytes. The main reason for these differences is that the metastasis of osteosarcoma is not a nonlinear and simple late event, but a dynamic and multimodal complex evolutionary process [9]. Moreover, the founder cells of the metastatic foci of bone and flesh were isolated from the primary foci very early and evolved independently under different evolutionary pressures. This may also explain why in some cases, early clonal events (such as ATRX mutations) are critical for subsequent metastasis initiation.

Metastatic progression is orchestrated by a network of soluble regulators and signaling axes, including C-C Motif Chemokine Ligand 5 (CCL5), C-X-C Motif Chemokine Ligand 12(CXCL12/SDF-1), Runt-Related Transcription Factor 2 (RUNX2), Transforming Growth Factor-beta (TGF-β), Interleukin-6 (IL-6), Vascular Endothelial Growth Factor (VEGF), and Nerve Growth Factor (NGF), that collectively drive tumor cell survival, invasion, immune evasion, angiogenesis, and neural remodeling [[10], [11], [12]]. Within the pulmonary niche, metastatic OS cells interact dynamically with endothelial cells, fibroblasts, immune cells, and neural components, forming a permissive microenvironment that supports metastatic outgrowth while simultaneously impeding drug penetration [3,[13], [14], [15]]. These processes are further reinforced by marked tumor heterogeneity, immune suppression, and tumor-nerve axis activation, rendering conventional systemic chemotherapy largely ineffective against lung metastases [[16], [17], [18], [19]]. Despite these insights, current clinical management remains heavily reliant on aggressive surgery and systemic chemotherapy, which face significant bottlenecks, including systemic toxicity limits the maximum tolerated dose, while the unique pulmonary hemodynamics and vascular barriers often result in insufficient drug accumulation and rapid clearance within the metastatic niche.

At the cellular level, metastatic OS cells and their supporting stromal components overexpress a series of targetable surface receptors, including Epidermal Growth Factor Receptor (EGFR), Astrocyte Elevated Gene-1 (AEG-1), Integrin alpha-V beta-3 (integrin αvβ3), Vascular Endothelial Growth Factor Receptor (VEGFR), and Cluster of Differentiation 133(CD133), which are implicated in metastatic homing, stemness maintenance, angiogenesis, and therapy resistance [[20], [21], [22]]. These receptors not only define the biological identity of LM-OS but also provide actionable entry points for precision therapeutic intervention. However, receptor expression in LM-OS is highly heterogeneous across patients, lesions, and disease stages, and even within individual pulmonary metastases [[23], [24], [25]]. As a result, traditional small-molecule or antibody-based approaches have struggled to exploit these targets effectively due to unfavorable pharmacokinetics, off-target toxicity, limited intratumoral accumulation, and insufficient coverage of heterogeneous metastatic cell populations [26,27].

These formidable clinical challenges create an urgent demand for nanoscale precision medicine. Traditional therapies lack the spatial precision required to distinguish between healthy lung parenchyma and metastatic nodules, as well as the temporal control necessary to sustain therapeutic concentrations amidst rapid pulmonary turnover. Nanomedicine, by virtue of its tunable physicochemical properties, offers a uniquely suited platform to overcome these multilevel biological barriers [28,29]. Ligand-modified nanoparticles enable receptor-specific uptake, while biomimetic strategies, such as cell membrane camouflage, enhance lung homing and immune evasion. Stimuli-responsive nanocarriers further exploit the acidic, hypoxic, or enzyme-rich metastatic microenvironment to achieve on-demand drug release [30,31]. Nevertheless, the effectiveness of nanomedicine in LM-OS is constrained by organ-specific delivery barriers, variable pulmonary vascular permeability, and rapid immune clearance, particularly in small or early-stage metastatic lesions. These considerations highlight the need for multifunctional yet disease-matched nanoplatforms capable of simultaneously addressing tumor heterogeneity, microenvironmental constraints, immune suppression, and tumor–nerve crosstalk [32].

Consistent with this concept, accumulating preclinical studies have demonstrated that precision nanomedicine can suppress LM-OS when delivery design is aligned with metastasis-relevant biology [33]. Representative strategies include EGFR- or integrin-guided nanocarriers that enhance selective uptake by metastatic and stem-like OS cells; hyaluronic acid-based nanoplatforms that enable CD44-associated, microenvironment- or intracellular-responsive drug release; and biomimetic or extracellular vesicle-inspired nanoparticles that improve pulmonary accumulation and metastatic niche targeting [[34], [35], [36]]. In parallel, immunomodulatory nanomedicines have been shown to reprogram the lung metastatic microenvironment and restore antitumor immunity beyond direct cytotoxic effects [37,38]. However, most reported successes remain confined to preclinical models, and therapeutic performance is often model- and context-dependent. Clinically relevant precedents, such as the liposomal immunomodulator mifamurtide evaluated in high-risk osteosarcoma and inhaled lipid-based cisplatin formulations tested in patients with pulmonary metastases, support translational feasibility, while also highlighting the substantial gap between experimental promise and routine clinical implementation.

**Fig. 1:**
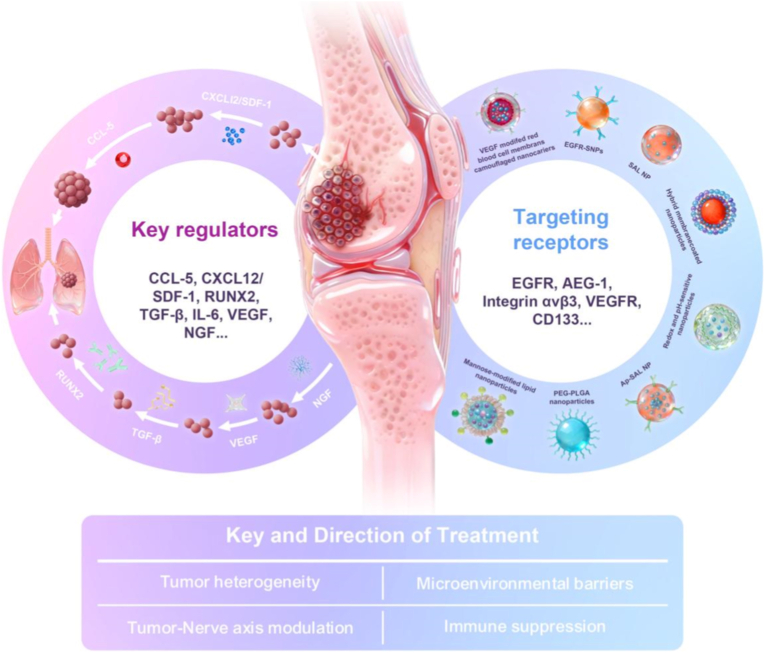
Biological drivers and precision nanomedicine intervention framework for lung metastatic osteosarcoma. Schematic overview of key molecular regulators driving osteosarcoma lung metastasis (left), clinically relevant targeting receptors exploited for precision nanotherapeutic delivery (right), and the corresponding therapeutic directions (bottom), including tumor heterogeneity management, microenvironmental barrier penetration, tumor-nerve axis modulation, and immune suppression reversal.

In this review, we focus specifically on precision nanomedicine strategies for LM-OS, adopting a barrier-oriented and disease-matched framework. As illustrated in Fig. 1, we first outline the key molecular regulators and signaling networks that govern OS lung metastasis, followed by an analysis of clinically relevant targeting receptors that can be exploited for precision delivery. We then systematically categorize nanotherapeutic strategies according to the principal therapeutic demands of LM-OS, including the management of tumor heterogeneity, penetration of pulmonary microenvironmental barriers, modulation of immunological and neural crosstalk, and reversal of metastatic immune suppression. Finally, we critically discuss the major translational challenges that currently constrain clinical application, such as biological heterogeneity, safety and clearance, manufacturing scalability, and the lack of predictive preclinical models, and highlight emerging prospects, including biomimetic and adaptive nanomedicine, gene editing and mRNA-based therapies, neuro-targeted intervention, and theranostic and AI-driven personalization. By anchoring nanomaterial design to the biological logic and metastatic context of LM-OS, this review aims to provide a rational roadmap for developing clinically translatable precision nanotherapies that improve both survival and quality of life for patients with this devastating disease.

## 2 Biological classification and metastatic microenvironment of lung metastatic osteosarcoma

LM-OS represents a biologically distinct and clinically lethal disease state rather than a simple extension of primary osteosarcoma. Although osteosarcoma originates from primitive bone-forming mesenchymal cells and exhibits substantial histological heterogeneity at the primary site, including osteoblastic, chondroblastic, fibroblastic, and surface variants, these classifications primarily describe local differentiation patterns and have limited predictive value for metastatic behavior [39]. In contrast, lung metastasis constitutes a convergent clinical endpoint shared across histological subtypes and defines patient prognosis, underscoring the need to conceptualize LM-OS as a systemic, metastasis-driven disease entity [25,40].

### 2.1 Metastatic competence and lung tropism of osteosarcoma

The propensity of osteosarcoma to metastasize to the lung is established early in disease progression [25]. Even at initial diagnosis, a substantial proportion of patients are believed to harbor occult pulmonary micrometastases, highlighting the inadequacy of viewing osteosarcoma as a localized bone malignancy. Metastatic competence is acquired through coordinated genetic, epigenetic, and microenvironmental programs that enable tumor cells to invade surrounding tissues, intravasate into the circulation, survive hemodynamic stress and immune surveillance, and ultimately colonize the pulmonary niche [3,13].

Key molecular regulators, including RUNX2, CXCL12/SDF-1, CCL5, TGF-β, IL-6, and VEGF, play central roles in this process by integrating osteogenic transcriptional programs with pro-invasive, pro-angiogenic, and immunomodulatory signaling [10,[41], [42], [43], [44], [45]]. These pathways endow osteosarcoma cells with enhanced motility, survival, and adaptability, while simultaneously shaping the stromal and immune landscape encountered at metastatic sites. The lung, characterized by its dense capillary network and first-pass filtration of venous blood, serves as the dominant site for metastatic arrest and colonization [46].

### 2.2 LM-OS microenvironment

Upon dissemination to the lung, osteosarcoma cells encounter a microenvironment that is fundamentally distinct from the bone niche. The pulmonary metastatic milieu comprises endothelial cells, fibroblasts, alveolar macrophages, and diverse immune populations, which collectively regulate metastatic fate [47]. Early disseminated tumor cells may undergo apoptosis or dormancy; however, permissive signaling cues can support their survival and eventual outgrowth into overt metastases [48].

Inflammatory and fibrotic remodeling of the lung microenvironment plays a critical role in this transition. Pro-metastatic cytokines such as TGF-β and IL-6 promote tumor cell survival, epithelial-mesenchymal plasticity, and angiogenesis, while abnormal vascular architecture and extracellular matrix deposition impose both physical and biological constraints on therapeutic access [[49], [50], [51]]. Importantly, immune suppression is a defining feature of established LM-OS lesions. The accumulation of regulatory T cells, myeloid-derived suppressor cells, and alternatively activated macrophages attenuates cytotoxic T lymphocyte and natural killer cell activity, facilitating immune escape and metastatic persistence [47].

### 2.3 Bone-lung-neural axis in osteosarcoma metastasis

Although lung metastases represent the terminal and lethal manifestation of osteosarcoma, their biology remains tightly coupled to the primary bone microenvironment. At the primary site, osteosarcoma disrupts bone homeostasis by promoting osteoclast-mediated bone resorption through Receptor Activator of Nuclear Factor-κB Ligand (RANKL) and parathyroid hormone-related peptide signaling. This process releases growth factors such as TGF-β and insulin-like growth factors from the bone matrix, further enhancing tumor aggressiveness and metastatic potential [52,53].

The neural regulatory network of LM-OS is a new interdisciplinary frontier field, namely “cancer neuroscience” [54]. Research in this field is revealing that tumors do not grow in isolation, but can hijack and remodel the host's neural signaling pathways, forming a bidirectional regulatory network, thereby driving their own proliferation, invasion, immune escape and colonization in the lung.

Osteosarcoma cells can not only produce neurotrophic factors autonomously, but also widely express corresponding receptors, forming multiple signal axes driving metastasis through autocrine and paracrine mechanisms. These include neurotrophins axis: NGF-TrkA, BDNF-TrkB and GDNF-RET, neuropeptide signal axis: substance P- NK-1R and CGRP-CRLR, and sympathetic signal: norepinephrine-β-adrenergic receptor [55]. Among these signaling axes, NGF-TrkA neural axis has been directly shown to significantly promote the migration and lung metastasis of osteosarcoma cells by upregulating the expression of matrix metalloproteinase-2 (MMP-2). NGF-TrkA signaling promotes neurite sprouting and activates downstream Phosphoinositide 3-Kinase-AKT Serine (PI3K-AKT), Mitogen-Activated Protein Kinase (MAPK), and Janus Kinase-Signal Transducer and Activator of Transcription (JAK-STAT) pathways, driving tumor proliferation, invasion, angiogenesis, and matrix remodeling [56,57].

Beyond its role in tumor progression, this tumor-nerve axis contributes to cancer-related bone pain and may prime osteosarcoma cells for metastatic dissemination, establishing a functional link between neural regulation at the primary site and lung metastatic competence [58,59]. The CGRP-NGF-TrkA loop releases CGRP through sensory nerve terminals to stimulate osteosarcoma cells to produce and release NGF. On the one hand, NGF promotes the proliferation and metastasis of tumor cells, on the other hand, it acts inversely on sensory nerve endings, resulting in the aggravation of pain, which forms a malignant positive feedback cycle of pain nerve activation NGF/CGRP release tumor progression pain aggravation, promoting the progression and metastasis of osteosarcoma.

**Fig. 2:**
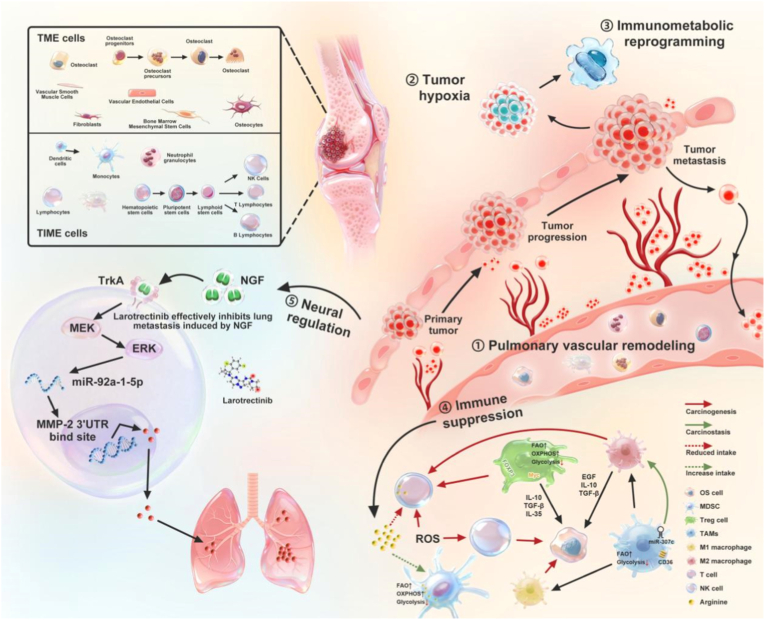
System-level biological landscape of lung metastatic osteosarcoma. Schematic illustration of the multiscale and multiorgan biological processes underlying lung metastatic osteosarcoma (LM-OS). Osteosarcoma originates from a heterogeneous bone microenvironment composed of osteogenic, stromal, vascular, immune, and neural components, and acquires metastatic competence through coordinated bone-tumor interactions. Primary tumors disseminate via the circulation to the lung, where metastatic colonization is shaped by pulmonary vascular remodeling, hypoxia, immunometabolic reprogramming, and immune suppression. In parallel, neural regulation, exemplified by the NGF-TrkA signaling axis, links tumor invasion, angiogenesis, immune modulation, and cancer-associated pain, forming a bone-lung-immune-neural crosstalk network that defines the biological context of LM-OS.

Collectively, these features define LM-OS as a biologically complex and therapeutically refractory disease state that cannot be adequately addressed by conventional cytotoxic approaches (Fig. 2). Recognizing LM-OS as a distinct metastatic entity, with unique molecular drivers, stromal interactions, and immune-neural regulation, is a prerequisite for the rational development of precision therapeutic strategies, including nanomedicine-based interventions designed to engage metastatic-specific vulnerabilities.

These intrinsic biological characteristics greatly increase the challenges encountered in the clinical management of LM-OS. Tumor heterogeneity is characterized by genetic alterations in different receptor profiles and different tumor cell subsets, which complicates treatment responses and hinders the efficacy of conventional treatment. In addition, the activation of immune evasion pathways, such as the overexpression of immune checkpoint inhibitors such as PD-L1, enables tumors to evade immune surveillance, thus limiting the effectiveness of immunotherapeutic approaches. The next chapter will specifically discuss the treatment barriers caused by these biological characteristics. Specifically, the limitations of current therapeutic strategies highlight the need for innovative approaches such as nanomedicine. Nanomedicines offer the potential to overcome these biological barriers by accurately targeting tumor cells, improving drug delivery efficiency and circumventing drug resistance mechanisms, thus providing a promising way for more effective treatment of LM-OS.

## 3 Therapeutic barriers and design challenges in LM-OS

Despite increasing recognition of LM-OS as a distinct disease entity, effective treatment remains elusive due to a series of interrelated biological and therapeutic barriers. Unlike primary tumors, LM-OS is characterized by pronounced cellular heterogeneity, metastatic site-specific receptor reprogramming, hostile pulmonary microenvironments, immune suppression, and tumor-nerve interactions [4,15,60]. These features collectively limit the efficacy of conventional systemic therapies and impose stringent constraints on precision treatment design. This section focuses not on redefining the biology of LM-OS, but on identifying the practical barriers encountered during therapy and clarifying the design requirements that next-generation nanomedicine must satisfy.

### 3.1 Tumor heterogeneity as a barrier to precision intervention

A defining challenge in LM-OS therapy is extreme tumor heterogeneity, which manifests at genetic, transcriptional, phenotypic, and functional levels. Metastatic lesions often comprise multiple subpopulations with distinct differentiation states, stem-like features, and drug resistance profiles. Single-cell transcriptomic analyses have revealed that malignant osteosarcoma cells segregate into multiple subclusters with divergent receptor expression and signaling dependencies, even within the same metastatic lesion [40,61]. As a result, therapeutic strategies targeting a single pathway or cell population frequently fail to achieve durable responses.

From a treatment perspective, tumor heterogeneity represents a design constraint rather than a biological descriptor. It undermines the efficacy of mono-target drugs and promotes rapid adaptive resistance following selective pressure [62]. For nanomedicine, this necessitates delivery systems capable of either engaging multiple targets simultaneously or dynamically adapting to heterogeneous tumor cell populations [63]. Accordingly, LM-OS heterogeneity demands multi-receptor targeting, combinatorial payload delivery, or programmable nanoplatforms that can maintain therapeutic coverage across diverse metastatic cell states.

### 3.2 Receptor reprogramming and limitations of single-target strategies

A second major barrier arises from differential receptor expression between primary osteosarcoma lesions and pulmonary metastases. During metastatic progression, osteosarcoma cells undergo receptor reprogramming that facilitates dissemination, survival, and colonization within the lung [64,65]. Upregulation of metastasis-associated receptors (e.g., c-Met, RANKL, CD47) and downregulation or redistribution of other signaling receptors have been observed in lung metastatic lesions, reflecting functional adaptation to the pulmonary niche [11,20,66,67].

This dynamic receptor landscape complicates precision therapy, as targeting strategies optimized for primary tumors may lose efficacy in metastatic sites [68,69]. Moreover, the coexistence of multiple receptor-defined subpopulations within LM-OS further limits the effectiveness of single-ligand or single-receptor targeting approaches [70]. For nanomedicine design, these observations argue against static, single-target paradigms and instead support modular, multivalent, or environment-responsive targeting strategies that can accommodate receptor heterogeneity and metastatic evolution.

### 3.3 Pulmonary microenvironmental barriers to drug delivery

The lung metastatic microenvironment presents formidable physical and biological obstacles to therapeutic delivery. Pulmonary metastases are often embedded within regions of abnormal vasculature, fibrosis, and extracellular matrix deposition, which collectively restrict vascular permeability, nanoparticle extravasation, and intratumoral penetration [13,52,64,71]. Vascular dysfunction characterized by altered VEGF signaling, imbalanced angiopoietin expression, and reduced adhesion molecule availability, further impairs immune cell trafficking and drug access [72,73].

Hypoxia represents an additional layer of complexity. Stabilization of hypoxia-inducible factors in LM-OS promotes angiogenic remodeling, epithelial-mesenchymal plasticity, metabolic reprogramming, and resistance to cytotoxic agents [52,74]. These hypoxic niches not only drive metastatic progression but also diminish therapeutic efficacy by limiting drug diffusion and altering cellular sensitivity [75,76]. From a design standpoint, effective nanomedicine systems for LM-OS must therefore be capable of overcoming diffusion barriers, responding to hypoxic or fibrotic microenvironments, and maintaining functional delivery within poorly perfused metastatic lesions.

### 3.4 Immune suppression as a therapeutic bottleneck

Immune suppression is a hallmark of LM-OS and constitutes a major barrier to both cytotoxic and immunotherapeutic interventions [77,78]. The pulmonary metastatic niche is enriched in regulatory T cells, myeloid-derived suppressor cells, and immunosuppressive macrophage populations that collectively inhibit cytotoxic T lymphocyte and natural killer cell activity [79,80]. This immune-excluded or immune-dysfunctional state enables metastatic persistence and undermines emerging immunotherapies.

From a therapeutic standpoint, immune suppression in LM-OS imposes a structural constraint rather than a single mechanistic defect [79]. It not only weakens endogenous antitumor immune surveillance, but also undermines the performance of treatment modalities that depend on immune engagement, such as antibody-based therapies and immune checkpoint blockade [81,82]. Nanomedicine-based approaches have therefore been explored as a means to reshape the metastatic immune landscape, for example by modulating suppressive myeloid populations, enhancing antigen presentation, or confining immune activation to metastatic sites [[83], [84], [85]]. Nevertheless, immune-targeted nanotherapeutic strategies operate within a narrow therapeutic window, where excessive or systemic immune stimulation may offset antitumor benefit with unacceptable toxicity, necessitating careful spatial and temporal control of immune modulation [86,87].

### 3.5 Tumor-nerve interactions and dual therapeutic constraints

Beyond classical tumor-immune interactions, neural regulation represents an underappreciated yet clinically relevant barrier in LM-OS therapy. The NGF/TrkA signaling axis links tumor progression, angiogenesis, immune modulation, and cancer-associated pain [88]. Activation of this pathway enhances metastatic aggressiveness while simultaneously contributing to debilitating bone pain, which complicates treatment tolerance and patient quality of life [89,90].

Targeting the tumor-nerve axis introduces unique therapeutic challenges. While inhibition of NGF/TrkA signaling can suppress tumor growth and alleviate pain [91,92], pathway redundancy, compensatory signaling, and acquired resistance limit the durability of single-agent interventions. For nanomedicine, this creates a demand for dual-purpose strategies capable of modulating neural signaling while concurrently delivering antitumor payloads, ideally with spatial and temporal precision to minimize off-target effects [93].

Collectively, as shown in Table 1, the barriers outlined above, tumor heterogeneity, receptor reprogramming, pulmonary microenvironmental constraints, immune suppression, and tumor-nerve crosstalk, define the therapeutic landscape of LM-OS. These challenges explain the limited success of conventional systemic therapies and underscore the need for advanced delivery paradigms [94,95].

**Table 1:** Therapeutic barriers in lung metastatic osteosarcoma and implications for precision nanomedicine design.

Therapeutic barrier	Biological basis in LM-OS	Impact on treatment efficacy	Implications for nanomedicine design
Tumor heterogeneity	Coexistence of stem-like and differentiated metastatic subpopulations with distinct receptor and signaling profiles	Single-target therapies show limited and short-lived efficacy	Multivalent, combinatorial, or adaptive nanoplatforms capable of addressing heterogeneous cell states
Receptor reprogramming	Dynamic alteration of receptor expression between primary tumors and lung metastases	Loss of efficacy of primary-tumor-oriented targeting strategies	Modular or multi-receptor targeting systems responsive to metastatic context
Pulmonary microenvironmental barriers	Abnormal vasculature, fibrosis, hypoxia, and dense extracellular matrix in lung metastases	Impaired drug extravasation, penetration, and intratumoral distribution	Microenvironment-responsive, penetration-enhanced, or stimuli-activated nanocarriers
Immune suppression	Enrichment of Tregs, MDSCs, and immunosuppressive macrophages in metastatic niches	Reduced efficacy of cytotoxic and immune-mediated therapies	Nano-immunomodulatory systems to reprogram immune microenvironment locally
Tumor-nerve interactions	NGF-TrkA-mediated neural remodeling and pain signaling	Enhanced tumor progression and limited treatment tolerance	Neuro-targeted or dual-purpose nanomedicines addressing both tumor growth and pain

For precision nanomedicine, LM-OS represents a disease context in which design must be explicitly informed by metastatic biology. Effective nanoplatforms must integrate multivalent or adaptive targeting, penetrate hostile pulmonary microenvironments, modulate immune and neural interactions, and accommodate dynamic tumor evolution. In the following sections, we discuss how contemporary nanomedicine strategies have been engineered to address these barriers and evaluate their potential for clinical translation in the management of lung metastatic osteosarcoma.

## 4 Specific nanocarrier platforms for the treatment of LM-OS

**Fig. 3:**
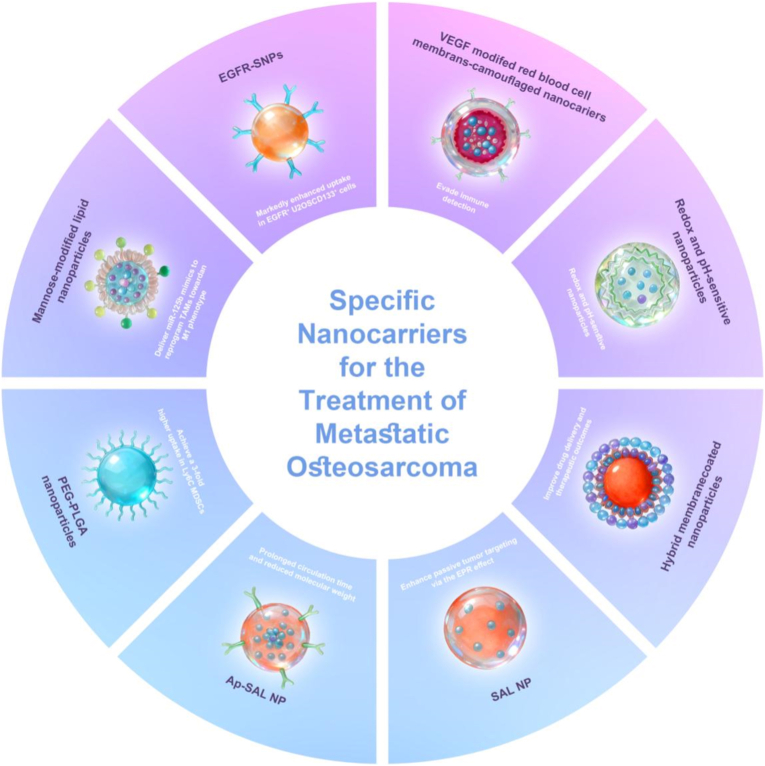
Representative nanocarrier platforms for lung metastatic osteosarcoma. Overview of disease-matched nanocarrier archetypes developed for metastatic osteosarcoma, including receptor-targeted, biomimetic/membrane-camouflaged, stimuli-responsive, and long-circulating platforms, illustrating platform-level strategies to address metastatic heterogeneity, pulmonary immune clearance, and microenvironment-dependent therapeutic access.

Guided by the therapeutic barriers outlined in Section 3, nanomedicine efforts in LM-OS have increasingly focused on platform-level solutions that can translate metastatic constraints into actionable delivery functions. In this section, we organize the literature by nanocarrier platform archetypes, rather than by material class or individual payload, because platform design ultimately determines how therapeutics navigate key LM-OS constraints [96,97](e.g., heterogeneous target engagement, pulmonary immune clearance, microenvironment-triggered activation, and access to disseminated lesions). Accordingly, we summarize representative platform categories that recur across LM-OS studies, including receptor-targeted nanocarriers [98], biomimetic/membrane-camouflaged systems, stimuli-responsive carriers, and long-circulating formulations [99] (Fig. 3).

While most LM-OS nanocarrier studies remain predominantly preclinical, their development trajectory shows a clear evolution from “delivery feasibility” toward metastasis-oriented functionality. Early work largely emphasized improving drug accumulation and cytotoxicity [100], whereas more recent platforms are designed to (i) broaden effective target coverage in heterogeneous metastatic lesions [101], (ii) mitigate rapid pulmonary immune clearance and improve metastatic interception, and/or (iii) exploit niche-associated cues to activate payload release locally. Taken together, current nanocarrier platforms for LM-OS primarily address metastatic complexity by either expanding target coverage [102], enhancing persistence and selectivity within the pulmonary niche [103], or enabling context-dependent payload activation [104], with different platform archetypes emphasizing distinct aspects of these objectives; the following sections examine these strategies in detail through representative LM-OS-relevant nanocarrier systems.

### 4.1 Tumour heterogeneity-oriented nanocarriers

Extensive intra- and inter-lesional heterogeneity is a defining feature of LM-OS [8]. Differences in receptor expression between primary bone lesions and pulmonary metastases, together with stem-like subpopulations that sustain relapse and therapeutic resistance, render single-target or single-mechanism interventions inherently insufficient. As discussed in Section 2, this heterogeneity is a biological property of the disease; here, the focus is specifically on how it constrains nanocarrier design and how platform-level strategies can be engineered to broaden effective target coverage and neutralize stem-associated vulnerabilities [105].

The multivalent targeting strategy in nanotechnology provides an idea for solving this problem. Its core is to use a nanocarrier to carry multiple or multispecific targeting ligands at the same time, so as to realize the extensive recognition and efficient combination of different subclonal cells in the tumor. The expression density of targets on tumor cell membrane is usually heterogeneous, especially in low expression subclones, a single ligand is difficult to bind efficiently. Multivalent targeting generates stronger overall binding force through the synergistic effect of multiple ligands on the surface of a nanoparticle and multiple targets, significantly improving the binding efficiency to tumor cells with low antigen expression. For example, in biomaterials, Wang et al. Significantly enhanced the recognition and killing of tumor cells with low expression antigen by constructing multivalent aptamer drug conjugates [106]. In addition, tumor heterogeneity includes the loss or downregulation of antigen expression, resulting in the failure of drugs that rely on a single target. Multivalent targeting endows nanoparticles with the ability to simultaneously recognize two or more different antigens on tumor cells [107]. Even if one target is lost, it can still be combined with other targets, effectively avoiding immune escape caused by antigen loss. The multivalent targeting strategy enables nanocarriers to simultaneously carry ligands targeting tumor cells and tumor endothelial cells (or stromal cells), achieving a coordinated attack on the entire tumor microenvironment [108].

#### 4.1.1 Receptor-landscape matching via multi-ligand and tunable targeting

One practical strategy to span heterogeneous receptor profiles is to engineer nanocarriers capable of engaging more than one surface marker and to tune ligand density according to the receptor landscape of a given model or patient sample [109]. Co-display of orthogonal ligands, such as aptamers, antibodies, or peptides, can increase the probability of productive binding across mixed tumour cell populations, while reducing reliance on any single receptor axis.

EGFR aptamer-functionalized salinomycin-loaded nanoparticles (EGFR-SNPs) provide a representative example of receptor-informed design in osteosarcoma [110]. Constructed as ∼90 nm polymer-lipid hybrid nanoparticles with approximately 8% drug loading and sustained release over five days, EGFR-SNPs achieved markedly enhanced uptake in EGFR^+^/CD133^+^ U2OS cells, reduced IC50 values by nearly four-fold relative to free salinomycin or non-targeted carriers, and significantly suppressed tumoursphere formation, indicating functional depletion of stem-like subpopulations. Importantly, this receptor-matched design translated into a measurable reduction in pulmonary metastatic burden in vivo, illustrating how even a single, well-chosen ligand can uncover functionally dominant metastatic subclones when heterogeneity is properly considered.

**Fig. 4:**
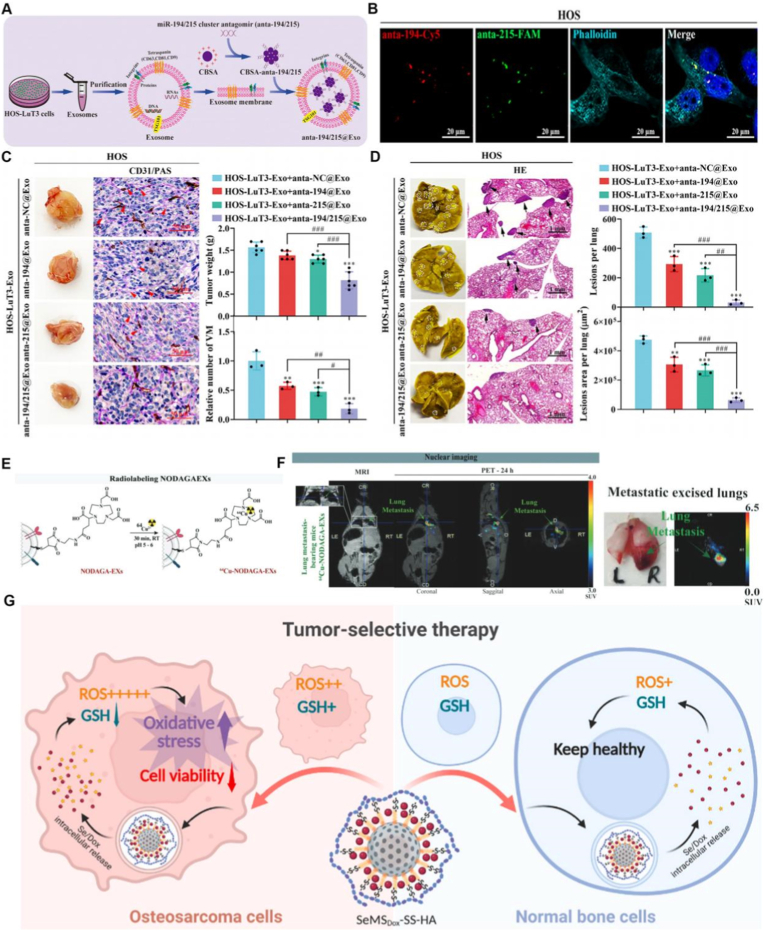
Tumour heterogeneity–oriented nanocarrier strategies for lung metastatic osteosarcoma. (A) Schematic illustration of exosomes isolated from lung metastatic HOS-LuT3 cells and engineered to co-deliver miR-194/215 antagomirs through membrane modification. (B) Confocal fluorescence imaging confirms efficient intracellular uptake and cytoplasmic colocalization of dual antagomirs in HOS cells. (C,D) Gross examination, CD31/PAS staining, and H&E analysis reveal significant suppression of lung metastatic burden, angiogenesis, and lesion area following dual-antagomir exosome treatment. Adapted from Ref. [113]. Copyright 2024, Elsevier B.V. (E,F) Radiolabeling and multimodal PET/MRI imaging further validate lung-selective accumulation and metastatic lesion visualization. Adapted from Ref. [114]. Copyright 2022, Wiley-VCH GmbH. (G) Schematic illustration of a tumor-selective, redox-responsive nanotherapeutic strategy, highlighting preferential intracellular drug activation in osteosarcoma cells with elevated ROS and GSH levels while sparing normal bone cells. Adapted from Ref. [115]. Copyright 2025, Elsevier Ltd.

Beyond single-receptor targeting, recent studies highlight the added value of multi-target or pathway-level coordination to suppress lung metastasis [111,112]. For example, exosome-based nanocarriers simultaneously delivering antagomirs against the miR-194/215 cluster were shown to significantly inhibit osteosarcoma lung metastasis by disrupting metastatic signaling at multiple regulatory nodes [113]. Fig. 4A demonstrates the construction and therapeutic evaluation of exosomes co-loaded with miR-194/215 antagomirs for osteosarcoma lung metastasis. Engineered exosomes derived from HOS-LuT3 cells enable stable encapsulation and co-delivery of dual antagomirs, which are efficiently internalized by HOS cells with clear cytoplasmic colocalization (Fig. 4B) [116]. In vivo imaging shows preferential pulmonary accumulation and prolonged retention of the dual-loaded exosomes. Therapeutically, treatment with miR-194/215 antagomir–loaded exosomes significantly reduces lung metastatic burden, suppresses tumor-associated angiogenesis, and decreases both the number and area of metastatic lesions compared with single-antagomir formulations, highlighting the advantage of coordinated multi-target interference in pulmonary metastatic osteosarcoma (Fig. 4C and D). This combinatorial strategy effectively suppressed metastatic outgrowth by concurrently targeting cooperative microRNA programs implicated in cell migration, survival, and colonization, underscoring the limitations of mono-target engagement in heterogeneous metastatic settings [114,117].

Building on this logic, dual-ligand designs, such as combining EGFR aptamers with CD44-binding hyaluronic acid shells, offer a route to simultaneously engage proliferative EGFR-high cells and more stem-like CD44-high compartments when co-expression is documented [118,119]. For such systems, controllable ligand density and spatial presentation are critical to avoid steric interference and maintain balanced engagement across targets [120,121].

#### 4.1.2 Preserving functional targeting through corona-aware and adaptive ligand exposure

Effective receptor targeting in vivo requires not only appropriate ligand selection but also preservation of ligand functionality throughout systemic circulation. Upon intravenous administration, nanoparticles rapidly adsorb serum proteins and form a protein corona [122], which can sterically shield or alter the presentation of surface ligands and thereby diminish receptor engagement, especially in heterogeneous metastatic settings. The dynamic and patient-specific nature of the protein corona further complicates targeting consistency across different metastatic niches.

To address this challenge, heterogeneity-aware nanocarriers increasingly incorporate corona-resistant interfaces and adaptive ligand exposure mechanisms. Dense PEG or zwitterionic coatings can reduce nonspecific protein adsorption in blood [123], while pH-labile or protease-cleavable linkers enable ligand unmasking within the acidic or enzyme-rich tumour milieu. This “mask-then-reveal” strategy allows the same nanocarrier to traverse circulation with minimal off-target interactions while restoring avid binding after extravasation into pulmonary metastases.

Although direct comparisons of such adaptive strategies in LM-OS models are still limited, analogous principles have already demonstrated efficacy in related osteosarcoma studies [124]. For example, osteosarcoma-derived small extracellular vesicles have been developed as natural homotypic carriers that intrinsically preserve surface markers and achieve preferential accumulation in pulmonary metastases (Fig. 4E and F), enabling sensitive detection and potential therapeutic delivery to micrometastatic lesions [114]. Similarly, cargo-eliminated osteosarcoma-derived vesicles engineered to compete for cellular uptake have shown reduced lung metastatic burden in preclinical models [125], highlighting how corona-resistant or biomimetic surfaces can preserve targeting functionality in the circulation and improve metastatic interception. Together, these examples underscore the importance of corona-aware design and adaptive ligand exposure for preserving functional targeting under physiological conditions. Systematic evaluation of such strategies in orthotopic and lung metastatic osteosarcoma models, preferably with quantitative analyses of corona composition and ligand accessibility, is warranted to translate receptor-directed nanomedicines into clinically effective therapies.

#### 4.1.3 Targeting stem-like phenotypes through payload selection and intracellularly triggered release

Because stem-like osteosarcoma cells underlie chemoresistance and metastatic persistence [126], heterogeneity-oriented nanocarrier design must extend beyond entry into the cell to include payload choice and intracellular release logic. Therefore, effective targeting of cancer stem-like compartments depends not only on surface phenotype recognition, but also on exploiting intracellular vulnerabilities that distinguish these cells from bulk tumor populations.

More broadly, intracellularly triggered release mechanisms can be leveraged to selectively activate cytotoxicity within stem-like compartments [127]. Disulfide-crosslinked nanocarriers that undergo reductive cleavage in the glutathione-rich cytosol are particularly well suited for this purpose. For instance, sarcoma-targeting peptide (STP)-decorated nanogels loaded with shikonin and crosslinked via reducible disulfide bonds achieved receptor-mediated uptake, on-site drug release, and significant suppression of both primary tumors and pulmonary metastases in orthotopic osteosarcoma models, with favourable tolerability [128]. By coupling surface phenotype recognition with intracellular vulnerability exploitation, such designs address heterogeneity at both the entry and execution stages, making them especially attractive for micrometastatic disease control.

Complementary strategies leverage dual endogenous stimuli to achieve even tighter control over intracellular activation [129]. For instance, pH- and redox-sensitive mesoporous silica nanoparticles incorporating selenium and doxorubicin enable site-specific drug release in the acidic, reduction-rich intracellular environment of osteosarcoma cells (Fig. 4G), enhancing cytotoxicity while sparing normal cells [115]. Such platforms illustrate how stimuli-responsive release mechanisms can be tuned to the intracellular milieu of resistant and stem-like cells, extending the design space beyond simple ligand-mediated targeting [130].

Collectively, these strategies illustrate that effective nanomedicine design for heterogeneous LM-OS requires coordinated control over target recognition, in vivo ligand functionality, and intracellular drug activation, rather than reliance on any single targeting element.

### 4.2 Bone and lung microenvironment-adaptive nanocarrier strategies

A defining challenge in LM-OS therapy lies in the necessity to simultaneously address two anatomically, mechanically, and immunologically distinct disease niches: the primary bone lesion and pulmonary metastatic sites. Unlike most solid tumors, osteosarcoma originates within a mineralized, mechanically rigid microenvironment [131] and subsequently disseminates to the lung [42], an organ characterized by dense capillary networks, continuous immune surveillance, and rapid clearance mechanisms. Consequently, nanocarrier platforms designed for LM-OS must accommodate dual-niche constraints, rather than being optimized for a single tumor context.

Rather than treating the bone and lung microenvironments as static delivery barriers, recent nanomedicine strategies increasingly leverage their unique physical, biochemical, and cellular features as design inputs, enabling enhanced retention, penetration, and site-selective drug activation [132]. Below, we discuss how nanocarrier platforms have been engineered to adapt to these divergent microenvironments and to coordinate therapeutic efficacy across primary and metastatic lesions.

#### 4.2.1 Size- and mechanics-adaptive nanocarriers for mineral-dense bone lesions

The osteosarcoma bone microenvironment is defined by extensive mineralization, dense extracellular matrix (ECM), and limited interstitial space, which collectively restrict nanoparticle diffusion and promote heterogeneous drug distribution. Conventional rigid or large-sized nanocarriers often exhibit poor penetration beyond perivascular regions [133], resulting in subtherapeutic exposure of deeply embedded tumor cells.

To overcome these limitations, size-adaptive nanocarriers have been developed that undergo controlled dimensional transformation after tumor entry [134]. For example, polymeric micelles or supramolecular assemblies that dissociate in response to tumor-associated enzymes or acidic pH [135] can transition from circulation-stable nanoparticles (∼70-100 nm) into smaller fragments (<40 nm), thereby enhancing diffusion through mineralized matrices. In osteosarcoma models, such systems have demonstrated significantly improved intratumoral penetration and more homogeneous drug distribution compared with static-size counterparts, translating into superior tumor growth inhibition. A representative example is the alendronate-triggered dual-cascade targeting prodrug nanoparticle strategy, which was explicitly designed to improve drug targeting and intratumoral penetration in osteosarcoma rather than only increasing bulk accumulation [136]; importantly, the authors emphasize penetration as a primary performance metric, consistent with the diffusion constraints imposed by mineral-dense lesions [137]. This type of “stable-to-penetrative” transition provides a workable design logic for bone lesions where static nanoparticles frequently stall at the tumor rim. In parallel, bone lesions offer a distinctive opportunity for bone-affinity–assisted retention coupled to deep access. For instance, an alendronate-based Pt(IV) prodrug amphiphile can self-assemble into lipid nanoparticles (APtIV) [136] that preferentially accumulate at osteosarcoma sites in vivo, and suppress primary tumor growth while alleviating bone destruction, an outcome profile that aligns with the dual requirement of tumor control and skeletal protection in OS management [136]. Although this platform is often discussed under “bone targeting,” its translational relevance to LM-OS stems from its potential to reduce the primary-tumor reservoir that continuously sheds metastatic precursors.

**Fig. 5:**
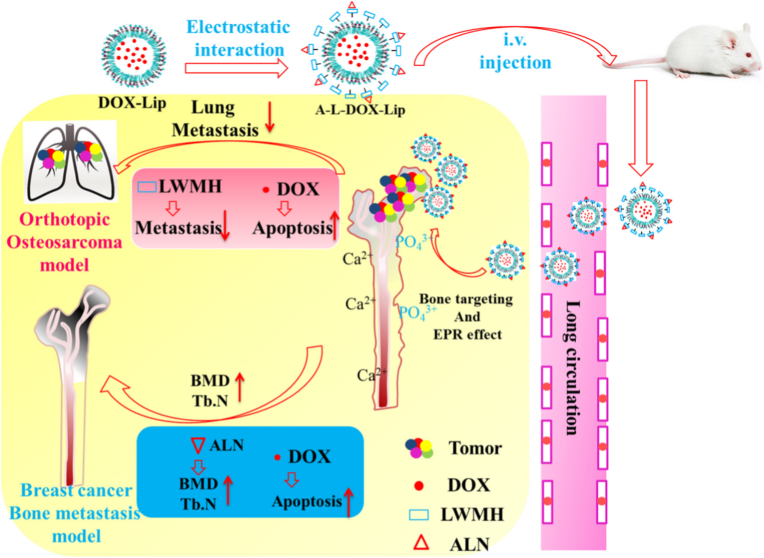
Schematic illustration of a bone-adaptive, mechanically compliant liposomal system for osteosarcoma treatment, showing prolonged circulation, enhanced penetration within the rigid bone microenvironment, and effective suppression of primary tumor growth and lung metastasis in an orthotopic osteosarcoma model. Adapted from Ref. [139]. Copyright 2020, Elsevier Ltd.

Beyond size, mechanical compliance (softness/deformability) is an under-quantified but increasingly relevant variable for navigating rigid, confined bone interstitium. While osteosarcoma-specific head-to-head datasets remain limited, the broader nanomedicine literature consistently shows that deformable particles can traverse tight pores more effectively than rigid counterparts, suggesting that mechanics-tuning should be treated as a first-class design parameter when penetration, not just accumulation, is the goal. In the osteosarcoma setting, related work using bone-targeting liposomal designs further supports the feasibility of building “bone-adaptive” carriers with improved in vivo performance [138]. As shown in Fig. 5, a bone-targeting liposomal system integrating mechanically compliant Low Molecular Weight Heparin (LMWH) coatings and mineral-affinitive ligands achieves prolonged circulation, efficient traversal of the rigid bone microenvironment, and enhanced intratumoral drug penetration, thereby suppressing primary osteosarcoma growth while markedly reducing downstream lung metastasis in an orthotopic model [139]. Going forward, rigorously measuring how particle modulus (and not only size) correlates with spatial drug distribution in orthotopic bone OS models will be essential, because penetration gains must be achieved without sacrificing circulation time or increasing off-target deposition.

#### 4.2.2 Pulmonary metastasis-oriented retention strategies

Beyond penetration, effective suppression of osteosarcoma lung metastasis requires not only delivery to the primary bone lesion but also durable retention within pulmonary metastatic niches. Prolonged residence can be achieved by leveraging site-specific biochemical interactions and clearance kinetics rather than relying solely on passive accumulation. Therefore, lung metastasis-oriented retention has emerged as a critical design dimension for nanocarriers intended to intercept disseminated tumor cells and established micrometastases.

**Fig. 6:**
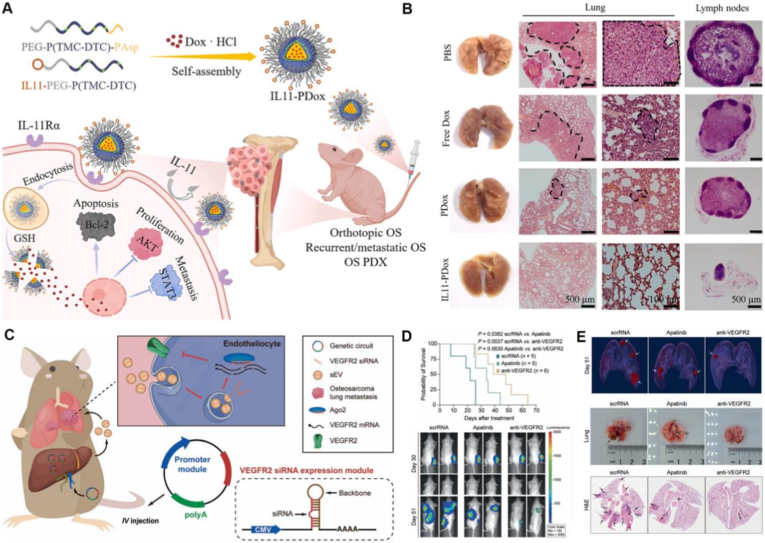
Pulmonary metastasis-oriented retention and microenvironment-adaptive nanostrategies for osteosarcoma. (A) Schematic illustration of pulmonary metastasis-oriented retention achieved by receptor-matched nanocarriers, exemplified by IL-11Rα–targeted polymeric assemblies that enhance accumulation and persistence within osteosarcoma lung metastatic nodules. (B) Representative gross images and histological analyses of lung and lymph node tissues showing effective suppression of pulmonary metastases through prolonged metastatic-site retention. (C) Illustration of microenvironment-adaptive delivery via in vivo self-assembled VEGFR2 siRNA-encapsulated small extracellular vesicles (sEVs), which exploit the pulmonary vascular niche for selective delivery to lung metastatic lesions. (D, E) In vivo imaging, survival analysis, and histopathological evaluation demonstrating that adaptation to the pulmonary metastatic microenvironment, rather than passive accumulation alone, confers superior inhibition of osteosarcoma lung metastasis. Adapted from Ref. [141]. Copyright 2023, Springer Nature.

A representative example of niche-matched retention is provided by IL-11Rα-targeted polymeric nanoparticles, which explicitly exploited receptor overexpression in osteosarcoma lung metastases [140]. In recurrent and patient-derived OS models, these nanoparticles achieved markedly higher accumulation and prolonged persistence within pulmonary metastatic nodules compared with non-targeted controls, resulting in near-complete suppression of lung metastases (Fig. 6A and B) [142]. Notably, comparable outcomes could not be achieved through passive targeting alone, underscoring that effective metastatic retention requires precise matching to lung-specific molecular cues rather than generalized circulation-based delivery [143].

Complementary to receptor-mediated strategies, modulation of clearance dynamics has also been shown to enhance pulmonary metastatic interception. Polyethylene Glycol (PEG)-containing lipid or polymeric nanoplatforms with optimized chain density reduced macrophage-mediated sequestration and delayed pulmonary clearance [144], thereby extending the window for interaction with metastatic lesions [145,146]. In osteosarcoma models, such circulation-persistent systems suppressed both intratibial primary tumor growth and lung metastasis, demonstrating that retention governed by clearance kinetics can directly translate into antimetastatic benefit. However, excessive PEG shielding was found to compromise extravasation efficiency, revealing an inherent trade-off between stealth and tissue access.

This limitation has motivated alternative retention paradigms that move beyond static stealth coatings. Recent lung metastasis-oriented biomimetic nanocarriers, including exosome-mimetic systems, have demonstrated preferential homing and sustained persistence at osteosarcoma pulmonary metastatic sites [114]. These designs highlight a shift toward metastatic-niche matching, in which retention is driven by site-specific biological recognition rather than passive Enhanced Permeability and Retention effect (EPR) or prolonged circulation alone [147]. Collectively, these studies establish pulmonary retention as an active, designable parameter that is indispensable for effective nanomedicine intervention against osteosarcoma lung metastasis.

#### 4.2.3 Adapting to the pulmonary metastatic microenvironment

In contrast to the bone niche, pulmonary metastases develop within a highly perfused and immunologically active organ. Although the extensive capillary network can facilitate nanoparticle access, rapid clearance by alveolar macrophages and lymphatic drainage severely limits nanoparticle persistence in metastatic lesions. Moreover, osteosarcoma tumor-derived factors actively remodel the lung microenvironment before overt metastasis, creating a pre-metastatic niche (PMN) characterized by stromal activation, extracellular matrix remodeling, and immunologic changes that facilitate metastatic colonization [148].

Nanocarrier designs for LM-OS therefore emphasize controlled pulmonary retention while minimizing immune activation, rather than relying on transient access alone [149]. Hydrodynamic size optimization plays a central role in this balance: particles within the ∼50-150 nm [150] range can transiently lodge within lung capillaries and enhance metastatic interception, whereas excessively small particles are rapidly cleared and larger particles are readily phagocytosed by macrophages. However, size alone rarely achieves durable retention once immune clearance mechanisms are engaged.

Beyond physicochemical tuning, microenvironment-matched biological delivery routes provide an alternative strategy to prolong pulmonary residence while preserving immune compatibility. A representative example is an in vivo self-assembly system in which intravenously administered genetic circuits reprogram the host liver to continuously secrete Vascular Endothelial Growth Factor Receptor 2 (VEGFR2) siRNA-encapsulated small extracellular vesicles (sEVs) [141]. Owing to their endogenous origin, optimal nanoscale size (∼100-150 nm), and natural tropism for highly perfused organs, these sEVs preferentially accumulated in osteosarcoma lung metastases and were efficiently internalized by pulmonary endothelial cells. Targeted silencing of VEGFR2 effectively suppressed pathological angiogenesis within metastatic lesions, leading to marked inhibition of lung metastatic progression and prolonged survival (Fig. 6), while avoiding the systemic toxicity commonly associated with small-molecule antiangiogenic agents.

This competitive uptake paradigm illustrates how microenvironment-matched biological interfaces can be exploited to extend pulmonary residence and functional influence without provoking overt immune responses [151]. In parallel, “stealth-to-active” [152] transitions such as environment-sensitive de-shielding (e.g., pH, enzyme, or redox triggers) may further enhance lesion localization by enabling circulation stability followed by selective activation upon arrival at metastatic sites. Collectively, these design strategies argue that pulmonary retention for metastatic interception must integrate immune compatibility, pre-metastatic niche modulation, and microenvironment-responsive activation as co-equal engineering axes for meaningful anti-metastatic efficacy in osteosarcoma.

#### 4.2.4 Overcoming stromal barriers through ECM-modulating nanocarriers

Both primary osteosarcoma lesions and pulmonary metastases frequently develop dense stromal compartments enriched in collagen, fibronectin, and proteoglycans. This ECM-rich architecture restricts nanoparticle diffusion and shields invasive tumor fronts from therapeutic exposure [153].

To address this barrier, nanocarriers incorporating ECM-responsive motifs have been developed. Matrix metalloproteinase (MMP)-cleavable peptide linkers, for example, can trigger de-shielding or payload release in protease-rich stroma; this is particularly relevant to osteosarcoma given that multiple MMPs (including gelatinases) are measurable and dynamically regulated during osteosarcoma progression [154,155].

The MMP active center contains a catalytic Zn ^2+^ ion that can recognize and cleave peptide bonds between specific amino acid residues in the peptide chain. It was found that the recognition of substrate peptides by MMP mainly depends on the properties of amino acid side chains at P1, P2, P1′, P2′ and other positions (where p represents the residue on the left side of the cleavage site and P′ represents the residue on the right side) [156]. In practice, enzyme-triggered “stealth-to-active” transitions (i.e., MMP-mediated cleavage of a protective layer) offer a rational route to enhance penetration while retaining circulation stability.

In parallel, ECM-modulating co-therapies seek to transiently loosen stromal architecture to improve transport. A representative and widely adopted strategy is to functionalize nanocarriers with collagenase to locally digest collagen networks, increasing intratumoral penetration and improving drug distribution, an approach with strong mechanistic plausibility for ECM-dense osteosarcoma lesions and pulmonary metastatic nodules, although careful dose and safety control is essential [157].

Other approaches combine mild stromal modulation with drug delivery, using low-dose collagenase or hyaluronidase to transiently loosen ECM architecture without inducing excessive tissue damage [158]. While such strategies require careful safety evaluation, they highlight the potential of controlled ECM remodeling to unlock otherwise inaccessible tumor regions.

#### 4.2.5 Integrating dual-niche targeting for systemic-metastatic synchronisation

Given that many osteosarcoma patients present with concurrent primary bone lesions and pulmonary metastases, carriers capable of coordinated adaptation to both bone and lung microenvironments [159] are an emerging design goal. Rather than treating these sites as separable therapeutic targets, recent work has explored dual-function nanocarriers [160] that integrate organ-specific targeting moieties with microenvironment-responsive modules to achieve compartment-adapted activities.

One class of such design leverages bone-targeting ligands (e.g., bisphosphonates) for primary lesions combined with stimuli-sensitive release mechanisms that can be activated under pathological conditions. For example, alendronate-functionalized, redox-sensitive liposomes loaded with chemotherapeutics have been developed to enhance bone accumulation via hydroxyapatite binding and improve intracellular release in reductive tumor environments; these dual-targeting liposomes not only exhibited enhanced growth inhibition in orthotopic osteosarcoma models but also reduced pulmonary metastases relative to non-targeted controls, illustrating the potential of combining bone affinity with microenvironment-responsive activation [161].

In addition to ligand-directed dual targeting, stimuli-responsive prodrug strategies provide a modular route to synchronise systemic and metastatic intervention. Although specific hypoxia-activated prodrugs have been applied in other tumor types to exploit microenvironmental differences between primary and metastatic sites, the overarching design paradigm, co-loading chemotherapeutics with environment-triggered prodrugs [162], offers a conceptual framework for future osteosarcoma metastasis-directed carriers.

Taken together, these studies demonstrate that microenvironment-adaptive nanocarrier strategies for LM-OS require fine-tuned coordination of size, surface chemistry, mechanical properties, and release behavior to navigate the fundamentally different constraints imposed by bone and lung niches. By transforming microenvironmental heterogeneity from a delivery obstacle into a design parameter, these platforms provide a mechanistic foundation for coordinated treatment of primary and metastatic osteosarcoma.

### 4.3 Immune suppression-oriented nanomedicine strategies in LM-OS

Immune evasion is a central hallmark of LM-OS, manifested by profound immunosuppression in both primary bone lesions and pulmonary metastases. Clinical and preclinical studies consistently report enrichment of myeloid-derived suppressor cells (MDSCs), M2-polarized tumor-associated macrophages (TAMs), and regulatory T cells (Tregs), together with elevated levels of inhibitory cytokines such as TGF-β and IL-10. These features collectively impair antigen presentation, suppress cytotoxic T lymphocyte (CTL) activity, and limit responsiveness to immune checkpoint blockade (ICB) [163,164]. In this context, nanomedicine strategies have been developed not simply to deliver immunotherapeutic agents, but to restructure the immune landscape of metastatic OS in a spatially and cell-selective manner.

#### 4.3.1 Nanocarrier-mediated modulation of immunosuppressive myeloid populations

Pulmonary metastatic osteosarcoma is characterized by a myeloid-dominated immunosuppressive microenvironment, in which M2-like TAMs and dysfunctional antigen-presenting cells collectively restrain cytotoxic T-cell activity and facilitate metastatic outgrowth [165]. Accumulating evidence suggests that effective immunotherapy in this setting does not primarily rely on immune cell depletion, but rather on functional reprogramming of immunosuppressive myeloid populations toward immune-supportive phenotypes [[166], [167], [168]]. Nanocarriers provide a unique opportunity to achieve this goal by integrating cell-selective delivery, pathway-level immune modulation, and spatiotemporally controlled release.

One representative strategy exploit macrophage-targeted drug co-delivery to rewire TAM polarization through coupled anti-angiogenic and metabolic intervention. Wang et al. developed mannosylated Poly(lactic-co-glycolic acid) (PLGA)-PEG nanoparticles for the co-delivery of regorafenib and α-difluoromethylornithine (DFMO), leveraging the overexpression of the mannose receptor Cluster of Differentiation (CD206) on M2-like TAMs and osteosarcoma cells. Rather than directly depleting macrophages, these nanocarriers induced robust TAM repolarization from an M2 to an M1 phenotype by simultaneously suppressing VEGF/VEGFR2-driven angiogenesis and inhibiting polyamine biosynthesis via Signal Transducer and Activator of Transcription 3 (STAT3) pathway attenuation [169]. This macrophage re-education was accompanied by increased CD8^+^ T-cell infiltration, reduced regulatory T cells, and a reshaped tumor immune microenvironment, ultimately leading to significant tumor growth suppression. Importantly, this study highlights that myeloid modulation can be achieved through pathway synergy rather than cytotoxic elimination, preserving immune homeostasis while restoring antitumor immunity.

**Fig. 7:**
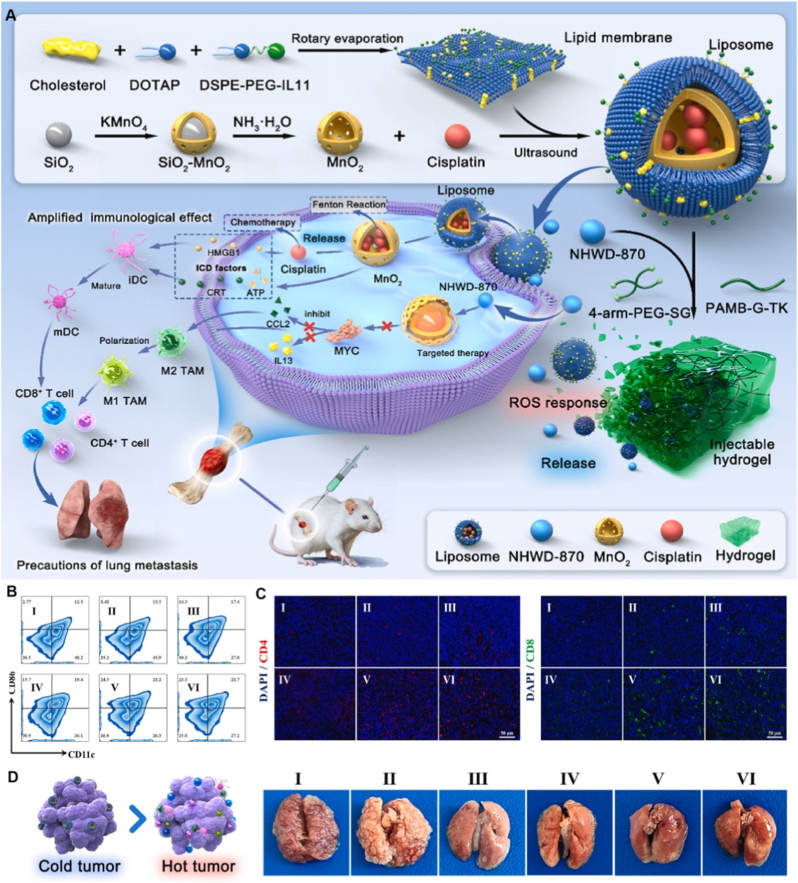
TME-responsive nanocomposite hydrogel–liposome platform for immunomodulatory osteosarcoma therapy. (A) Schematic illustration of a TME-responsive nanocomposite hydrogel–liposome system for osteosarcoma therapy. The platform integrates IL11Rα-targeted liposomes co-loaded with cisplatin and MnO_2_ and an MYC inhibitor (NHWD-870) within an injectable, ROS-responsive hydrogel, enabling localized, sustained release. (B) Flow cytometric analysis showing macrophage polarization changes, indicating a shift from immunosuppressive M2-like TAMs toward pro-inflammatory M1 phenotypes after treatment. (C) Immunofluorescence staining of tumor tissues demonstrating enhanced immunogenic cell death and increased infiltration of CD8^+^ T cells following nanocomposite hydrogel treatment. (D) Representative lung images illustrating effective suppression of pulmonary metastasis and conversion of immunologically “cold” tumors into “hot” tumors in treated osteosarcoma-bearing mice. I: Control, II: PGP, III: Mn@PGP, IV: Cis/Mn@PGP, V: Mn/NH@PGP, VI: Cis/Mn/NH@PGP. Adapted from Ref. [170]. Copyright 2025, Elsevier Ltd.

Complementary to macrophage-centered approaches, nanocarrier-enabled oncogene regulation coupled with immunogenic cell death (ICD) provides an additional axis for myeloid reprogramming. In a TME-responsive nanocomposite hydrogel system reported by Ma et al., an MYC inhibitor (NHWD-870) was combined with IL11Rα-targeted cisplatin/MnO_2_-loaded liposomes for MYC-amplified osteosarcoma [170]. MYC degradation suppressed tumor-derived cytokines such as CCL2 and IL-13, which are key drivers of M2 macrophage polarization, thereby promoting a shift toward an M1-like phenotype. Concurrently, MnO_2_-mediated ROS amplification and cisplatin-induced ICD triggered dendritic cell maturation and activation of the cGAS-STING pathway, linking tumor cell death to innate immune activation [171]. This dual myeloid modulation, macrophage repolarization and dendritic cell activation, resulted in enhanced T-cell infiltration and a marked reduction in pulmonary metastases, demonstrating that nanocarriers can orchestrate coordinated innate immune reprogramming by targeting both tumor-intrinsic and microenvironmental cues (Fig. 7).

Together, these principles highlight that nanocarrier-enabled immunotherapy for metastatic osteosarcoma should be conceived not as a delivery problem, but as a systems-level immune engineering task, in which carrier design, signaling pathway selection, and microenvironmental responsiveness are co-optimized to reprogram the myeloid compartment and unlock effective antitumor immunity.

#### 4.3.2 Nanoparticle-assisted enhancement of immune checkpoint blockade

Despite strong biological rationale, clinical responses to PD-1/PD-L1 blockade in osteosarcoma have been limited, particularly in metastatic disease [82]. Conventional immune checkpoint blockade (ICB) therapies targeting PD-1/PD-L1 can restore T-cell activity by blocking inhibitory signaling, yet this mechanism alone may be insufficient in a TME dominated by myeloid suppression, T-cell exhaustion, and heterogeneous checkpoint expression. As a result, there is an emerging focus on nanoparticle-mediated modulation of upstream immunosuppressive pathways to sensitize tumors to ICB.

A key upstream pathway implicated in both immune suppression and checkpoint regulation is the STAT3 signaling axis. STAT3 activation within tumor and immune cells can drive PD-L1 expression and promote an immunosuppressive milieu by enhancing IL-10 and VEGF production, thereby limiting T-cell infiltration and function [60]. Nanoparticle platforms that deliver STAT3 inhibitors or STAT3-targeting siRNA have been developed as a means to reduce STAT3 activity locally within the TME and improve immune responsiveness. For example, biomimetic polydopamine nanoparticles camouflaged with stem cell membranes have been used to co-deliver curcumin and siSTAT3 for synergistic osteosarcoma therapy, demonstrating effective delivery of STAT3-targeting nucleic acids and modulation of tumor growth in preclinical models [172]. While this work does not directly assess checkpoint blockade synergy, it provides a mechanistic foundation for how STAT3 inhibition via nanocarriers can alter the immunosuppressive environment that undermines ICB efficacy.

**Fig. 8:**
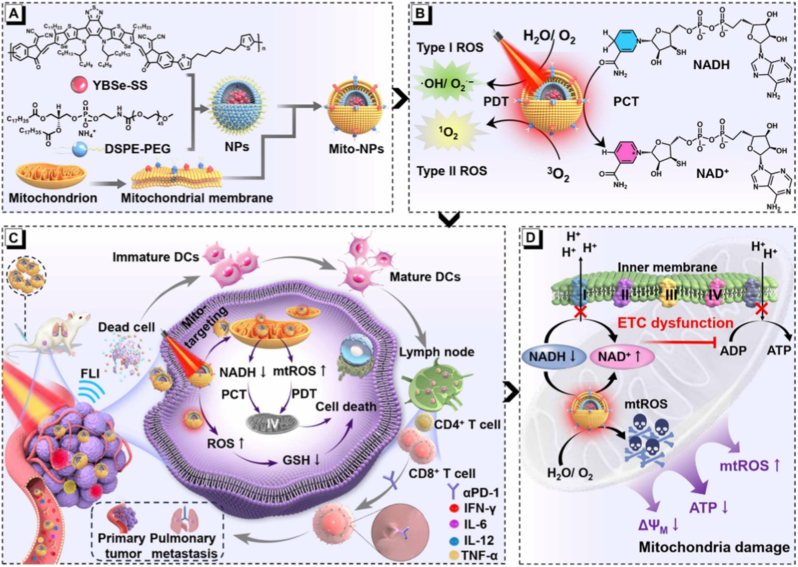
Nanoparticle-induced mitochondrial stress and immunogenic cell death for sensitizing immune checkpoint blockade. (A) Schematic illustration of mitochondria-targeted nanoparticles constructed via DSPE-PEG modification, enabling selective accumulation at the mitochondrial membrane. (B) Upon photoactivation, the nanoplatform simultaneously induces type I and type II reactive oxygen species (ROS) generation through photodynamic and photocatalytic processes, amplifying oxidative stress. (C) Excessive mitochondrial ROS disrupt redox homeostasis and trigger immunogenic cell death, characterized by calreticulin exposure, ATP and HMGB1 release, leading to dendritic cell maturation and subsequent activation of CD8^+^ T cells. (D) Mitochondrial electron transport chain dysfunction and collapse of mitochondrial membrane potential further exacerbate tumor cell death, converting immunologically “cold” tumors into “hot” tumors and enhancing responsiveness to PD-1/PD-L1 blockade. Adapted from Ref. [173]. Copyright 2025, AAAS.

In parallel to pathway-level immune de-repression, nanoparticle-enabled amplification of ICD and mitochondrial stress has emerged as another effective route to sensitize tumors to immune checkpoint blockade. A representative example involves mitochondria-targeted nanoparticles engineered to induce coordinated photodynamic therapy (PDT) and photothermal/photocatalytic therapy (PCT), thereby triggering excessive mitochondrial reactive oxygen species (mtROS) generation (Fig. 8) [173]. These nanoplatforms disrupt mitochondrial electron transport chain function, collapse the mitochondrial membrane potential, and deplete intracellular Nicotinamide Adenine Dinucleotide (NADH), leading to irreversible tumor cell death accompanied by robust ICD hallmarks, including calreticulin exposure, Adenosine Triphosphate (ATP) release, and High Mobility Group Box 1 (HMGB1) secretion. The resulting danger signals promote dendritic cell maturation and enhance antigen presentation within draining lymph nodes, ultimately increasing CD8^+^ T-cell priming and infiltration into metastatic lesions. By converting immunologically “cold” tumors into “hot” tumors through mitochondria-centered stress signaling, such nanocarrier-induced ICD markedly enhances the therapeutic responsiveness to PD-1/PD-L1 blockade, illustrating how organelle-targeted nanomedicine can function as an immune adjuvant that unlocks checkpoint efficacy.

Collectively, these nanomedicine approaches highlight a generalizable principle for enhancing ICB in osteosarcoma: nanoparticles can function as immune “primers” that modulate suppressive signaling networks, increase effector cell infiltration, and improve the local effectiveness of checkpoint blockade. By integrating upstream immunomodulation with targeted delivery and controlled release, nanoparticle-assisted ICB strategies offer a promising path to overcome the inherent resistance of osteosarcoma lesions to current immunotherapies.

#### 4.3.3 Nanoparticle-based cytokine delivery for localized immune activation

Cytokine therapy is a potent immunotherapeutic modality but is frequently constrained by systemic toxicity and a narrow therapeutic window, motivating the development of localized and controlled delivery strategies to improve safety while maintaining efficacy [174]. In LM-OS, nanocarrier-based cytokine delivery has been explored to achieve localized, sustained immune activation while avoiding systemic adverse effects.

A representative osteosarcoma-relevant example is the use of IL-12–loaded PLGA nanospheres, which were developed to enable pharmacologically meaningful IL-12 delivery while mitigating the toxicities observed with systemic recombinant IL-12 administration [175]. In metastatic osteosarcoma models, this formulation conceptually supports prolonged cytokine availability and immune stimulation (e.g., IFN-γ–linked antitumor responses) through a depot-like nanosphere release profile, illustrating how polymeric cytokine encapsulation can decouple efficacy from systemic exposure.

In parallel, lung-localized cytokine administration provides a clinically instructive route for LM-OS, leveraging the pulmonary delivery interface to confine bioactivity to metastatic lesions. Aerosolized liposomal IL-2 has been reported to be safe and effective in dogs with spontaneous pulmonary metastases from osteosarcoma, supporting the feasibility of lung-restricted cytokine delivery to enhance local immune activity while limiting systemic toxicity. Consistently, in murine models of osteosarcoma lung metastasis, aerosol IL-2 augmented NK-cell–based therapy and improved overall survival, further underscoring the value of spatially confined cytokine exposure for pulmonary metastatic control [176,177].

Collectively, these case-driven examples demonstrate that nanomedicine strategies targeting immune suppression in LM-OS operate through multiple, complementary mechanisms, including myeloid cell modulation, ICD induction, checkpoint sensitization, and localized cytokine delivery. While monotherapies targeting individual immune components can partially restore antitumor immunity, the redundancy and adaptability of the metastatic immune microenvironment suggest that integrated, multi-pronged nanoplatforms, potentially combined with ICB or personalized vaccines, will be required for durable disease control.

To facilitate a systematic understanding of how nanomedicine strategies address the multifaceted barriers underlying lung-metastatic osteosarcoma, Table 2 summarizes representative platform-level designs mapped to distinct biological constraints. These barriers span tumor heterogeneity, bone and pulmonary microenvironmental obstacles, and immune suppression, each of which necessitates tailored nanocarrier engineering rather than one-size-fits-all solutions. As highlighted, contemporary nanomedicine approaches leverage receptor matching, biomimetic interfaces, adaptive physicochemical properties, and microenvironment-responsive release to improve target coverage, tissue penetration, and lesion retention across heterogeneous metastatic niches. In parallel, immune-oriented platforms focus on myeloid reprogramming, checkpoint sensitization, and localized cytokine delivery to overcome the dominant immunosuppressive landscape of metastatic disease. Collectively, this framework illustrates that effective nanotherapeutic intervention in LM-OS increasingly relies on integrated, barrier-aware design principles, positioning nanocarriers as active modulators of tumor–microenvironment interactions rather than passive drug shuttles.

**Table 2:** Barrier-oriented nanomedicine strategies for lung metastatic osteosarcoma.

**Targeted Barrier**	**Nanomedicine Strategy (Platform Level)**	**Representative Mechanisms and Examples**
Tumor heterogeneity	Receptor-matched and multi-ligand nanocarriers	EGFR aptamer-salinomycin nanoparticles (EGFR-SNPs); dual-recognition systems combining EGFR aptamers with CD44-targeting hyaluronic acid shells to broaden target coverage across heterogeneous metastatic subpopulations
	Corona-aware and adaptive ligand-exposure interfaces	PEGylated, size-adaptive polymeric micelles enabling reduced opsonization during circulation; environmentally responsive ligand exposure; red blood cell membrane-camouflaged nanocarriers preserving targeting functionality in vivo
	Stem-like phenotype targeting with intracellularly triggered release	Salinomycin-loaded nanoparticles selectively eradicating CSC-like cells; disulfide-crosslinked nanogels loaded with shikonin enabling redox-triggered intracellular release; pH- and MMP-responsive nanoparticle systems
	Biomimetic interface nanocarriers	PLGA core nanoparticles cloaked with osteosarcoma cell or macrophage membranes; leukocyte-tumor hybrid membrane platforms (leutusomes) for enhanced recognition of heterogeneous tumor populations
Bone and lung microenvironmental barriers	Size-adaptive and stiffness-tuned nanocarriers	PEGylated polymeric micelles undergoing size reduction after tumor entry; moderately soft lipid-polymer hybrid nanoparticles facilitating penetration within mineral-dense bone matrices
	Bone-anchoring and bone-retentive nanocarriers	Bisphosphonate-functionalized liposomes loaded with doxorubicin; Asp_8_-guided polymeric nanoparticles exploiting calcium-rich resorption lacunae
	Pulmonary metastatic niche retention strategies	Aerodynamically optimized nanoparticles (50-150 nm) for enhanced lung interception; anti-IL-11Rα antibody-modified polymeric nanoparticles targeting lung metastatic lesions
	ECM-degradable and stromal-modulating carriers	Gelatinase-responsive liposomes encapsulating paclitaxel; mesoporous polydopamine nanoparticles combined with photothermal therapy and bromelain to enhance stromal penetration
	Dual-niche-adaptive integrated platforms	pH-sensitive, alendronate-decorated polymeric nanoparticles co-loaded with doxorubicin and hypoxia-activated prodrugs for coordinated control of bone lesions and lung metastases
Immune suppression	Myeloid cell depletion or reprogramming nanocarriers	Anti-Ly6C-modified PEG-PLGA nanoparticles co-delivering gemcitabine and STAT3 inhibitors for MDSC depletion; mannose-modified lipid nanoparticles delivering miR-125 b mimics to reprogram M2-like TAMs
	Checkpoint blockade-sensitizing nanocarriers	STAT3 siRNA nanoparticles reducing PD-L1 expression; polymeric micelles encapsulating IDO inhibitor NLG919; IL-11Rα-targeted nanostrategies enhancing response to immune checkpoint blockade
	Localized cytokine delivery systems	PLGA nanoparticles encapsulating IL-12 mRNA with anti-CD11c targeting; hydrogel-embedded liposomes enabling sustained IL-2 release.

## 5 Challenges

Despite substantial progress in nanocarrier design and encouraging preclinical results, the clinical translation of nanomedicine for LM-OS remains limited. The barriers to translation are not confined to any single aspect of formulation or delivery, but instead arise from the intrinsic complexity of the disease itself, spanning biological heterogeneity, microenvironmental constraints, immune and neural regulation, as well as safety and regulatory considerations. Importantly, many of these challenges are tightly interconnected: strategies that improve targeting precision may exacerbate manufacturing complexity, while approaches that enhance tissue penetration or immune activation may introduce new safety liabilities. In the following sections, we delineate the major challenges that currently impede the effective clinical deployment of nanomedicine in LM-OS, with a focus on how disease-specific features impose practical limits on otherwise rational design strategies.

### 5.1 Tumour heterogeneity and the limits of precision targeting

A central challenge in translating nanomedicine to LM-OS lies in the extreme spatial and temporal heterogeneity of the disease. Osteosarcoma exhibits pronounced interpatient variability, intratumoural diversity, and phenotypic divergence between primary bone lesions and pulmonary metastases. Within individual lesions, proliferative tumor cells coexist with quiescent, stem-like subpopulations that display enhanced drug resistance and metastatic competence. This complexity fundamentally constrains precision targeting strategies.

Most actively targeted nanocarriers rely on a limited set of surface receptors, such as EGFR, CD44, or IL-11Rα, whose expression levels vary not only among patients but also across metastatic nodules within the same lung. As a result, ligand-receptor matching optimized in one model may fail in another, and single-ligand systems risk selectively eliminating only a subset of malignant cells while sparing relapse-initiating populations. Moreover, receptor expression can evolve under therapeutic pressure, further eroding targeting fidelity over time.

An additional and often underestimated limitation arises from protein corona formation. In vivo adsorption of plasma proteins can obscure targeting ligands, alter surface charge, and redirect nanocarriers toward off-target organs, undermining carefully engineered specificity. Although multi-ligand designs and environment-responsive ligand exposure have been proposed to mitigate these effects, such approaches substantially increase formulation complexity, raise manufacturing challenges, and complicate regulatory characterization [178]. Consequently, balancing targeting breadth, specificity, and translational feasibility remains an unresolved issue in LM-OS nanomedicine.

### 5.2 Microenvironmental barriers and the unpredictability of delivery

The microenvironmental context of LM-OS presents another major translational barrier. Unlike many epithelial cancers, osteosarcoma arises in mineralized bone with limited perfusion and subsequently colonizes the lung, where metastatic lesions often exhibit compact architecture and intact endothelial barriers [39]. As a result, the enhanced permeability and retention (EPR) effect, frequently invoked to justify nanoparticle accumulation, proves highly inconsistent in LM-OS, particularly in pulmonary micrometastases.

In bone lesions, dense extracellular matrix, calcified structures, and elevated interstitial pressure restrict nanoparticle penetration beyond perivascular regions [179]. In the lung, although vascular access is readily achieved, rapid clearance by resident immune cells and lymphatic drainage limits nanoparticle retention. These contrasting constraints make it difficult to design a single carrier that performs optimally across both niches.

Strategies such as reducing particle size, engineering shape- or size-transformable carriers, incorporating penetration-enhancing peptides, or applying external stimuli (e.g., ultrasound) have shown promise in preclinical models [180]. However, deeper tissue penetration may also increase systemic dissemination, off-target accumulation, and toxicity. Importantly, the relationships between nanoparticle physicochemical properties and organ-specific vascular and stromal features remain incompletely understood, limiting the predictability of delivery outcomes and complicating rational design.

### 5.3 Immunological and neural interplay: efficacy versus safety

Immunological factors exert a profound influence on nanomedicine performance in LM-OS [181]. Pulmonary metastases are embedded within immunosuppressive niches enriched in regulatory T cells, myeloid-derived suppressor cells, and M2-like macrophages, which collectively dampen cytotoxic T-cell responses. Nanoparticles must therefore evade clearance by the mononuclear phagocyte system while, in some cases, actively reshaping the immune microenvironment.

This dual requirement creates a narrow therapeutic window. Incorporation of immunostimulatory agents, such as toll-like receptor agonists or STING activators, can enhance antitumor immunity but also carries a significant risk of systemic inflammation, cytokine release, and immune-related toxicity. In addition, nanomaterials themselves may activate complement pathways, induce cytokine secretion, or elicit anti-PEG antibodies, leading to accelerated blood clearance upon repeated dosing.

At the same time, in order to make nanomedicines circulate in the blood longer, polymers such as polyethylene glycol (PEG) are usually used for stealth coating modification [182]. However, the immune system can also recognize this foreign body. In order to achieve active targeting, the surface of nanoparticles will be connected with targeting ligands such as antibodies and peptides. These ligands (especially those derived from different species, such as humanized antibodies) may be recognized by the immune system, thereby inducing the production of targeted ligand specific antibodies. A study found that compared with free ligands, targeting ligands attached to nanoparticles showed higher immunogenicity and could induce higher antibody titers.

The anti nanobody produced by immunogenicity will accelerate blood clearance (ABC phenomenon) during repeated administration. Anti nanoantibodies (such as anti peg IgG) will bind to the reinjected nanomedicines, making them rapidly recognized and cleared by the immune system (especially macrophages in the liver) [183]. Studies have shown that this may lead to a 50-60% reduction in the circulation half-life and tumor accumulation of nanomedicines, while the accumulation in the liver increases by 50%. This not only greatly reduces the therapeutic effect, but also may increase hepatotoxicity.

An additional and largely underexplored dimension is the neural microenvironment. Neurotrophin-driven signaling, particularly the NGF-TrkA axis, promotes osteosarcoma growth, angiogenesis, pain sensitization, and metastatic progression. While this pathway represents an attractive therapeutic target, systemic inhibition of NGF or TrkA risks impairing nerve function and regeneration [56,184]. Nanomedicine offers a potential solution through spatially restricted delivery; however, achieving precise targeting of tumor-associated neural elements without affecting normal innervation remains technically challenging and insufficiently validated.

### 5.4 Safety, clearance, and manufacturing constraints

Safety considerations continue to pose a major hurdle for clinical translation. Many inorganic nanomaterials, including graphene derivatives and quantum dots, exhibit prolonged retention in the liver, spleen, and other organs, raising concerns regarding chronic toxicity, inflammation, and potential genotoxicity. Even biodegradable platforms such as PLGA or lipid nanoparticles may accumulate if degradation or clearance is incomplete, particularly under repeated dosing regimens.

Nanoparticles retained in the lungs will be phagocytosed by immune cells such as alveolar macrophages. If the particles are difficult to degrade, it will trigger destroyed phagocytosis, leading to the continuous release of inflammatory factors and reactive oxygen species by cells, triggering oxidative stress. These inflammatory mediators not only damage lung tissue, but also affect extrapulmonary organs such as cardiovascular system through blood circulation, resulting in systemic effects [185]. The half-life of different inorganic materials varies greatly. The half-life of gold-based materials is more than 672 h, while that of iron oxide (Fe_2_O_3_) only takes 3.6-3.91 days [186]. In addition, although silica nanoparticles (SiNPs) have been widely studied due to their good biocompatibility and modifiability, precise data on their lung clearance half-life are very limited, and the lung toxicity brought by their long-term exposure, especially the risk of inflammation and fibrosis, is the core obstacle for clinical application. Although silicon-based materials are considered safer than many metal nanomaterials (such as gold and silver), their long-term behavior and safety assessment in the lung are still complex.

Efforts to accelerate clearance, for example by reducing particle size or employing zwitterionic surfaces, may alleviate long-term retention but often come at the cost of reduced tumor accumulation and shortened circulation time. Striking an optimal balance between efficacy and clearance remains a persistent design challenge.

Manufacturing scalability and reproducibility further complicate translation. Many nanomedicines incorporate multiple components, targeting ligands, stimuli-responsive elements, immunomodulators, each introducing additional sources of variability. Batch-to-batch differences in size distribution, drug loading, or ligand density can significantly affect pharmacokinetics and therapeutic performance. Regulatory agencies require rigorous and standardized characterization of nanomedicines, including surface chemistry, stability, residual solvents, and endotoxin levels. High production costs associated with complex formulations or rare ligands may further limit clinical adoption.

### 5.5 Preclinical models and the translational gap

Finally, the gap between preclinical success and clinical efficacy remains a critical and unresolved challenge in the development of nanomedicine for LM-OS. Most nanotherapeutic strategies are initially evaluated in murine models, including subcutaneous or orthotopic xenografts and genetically engineered mouse models. While these systems are indispensable for mechanistic validation and early efficacy screening, they fail to fully recapitulate key features of human LM-OS, particularly the complexity of the immune system, the architecture of pulmonary metastases, and the tempo of metastatic progression. Consequently, therapeutic benefits observed in mice, especially dramatic reductions in metastatic burden, often overestimate clinical efficacy.

From a current practice perspective, the majority of LM-OS nanomedicine studies rely on immunodeficient mice bearing rapidly growing tumors, conditions that favor nanoparticle accumulation and therapeutic response but obscure immune-mediated clearance, toxicity, and long-term safety. Even in syngeneic models, murine immune systems differ substantially from those of humans in cytokine signaling, macrophage polarization, and complement activation, all of which directly influence nanoparticle fate. These discrepancies limit the predictive power of conventional mouse models for both efficacy and immunotoxicity, particularly for immunomodulatory and biomimetic nanoplatforms.

Large-animal models, most notably canine osteosarcoma, represent a more clinically relevant intermediate between murine studies and human trials [187,188]. Canine OS shares striking similarities with human disease in terms of genetic alterations, tumor heterogeneity, metastatic patterns, and immune contexture, and dogs develop spontaneous lung metastases under natural immune surveillance. Importantly, the reported efficacy of liposomal IL-2 therapy in dogs highlights the value of canine models for evaluating dosing regimens, biodistribution, immune activation, and safety at physiologically relevant scales [189]. At present, such models occupy a quasi-translational stage, bridging proof-of-concept studies and first-in-human trials. Nonetheless, species-specific differences in metabolism, immune regulation, and nanomaterial clearance persist, underscoring the need for cautious interpretation when extrapolating canine data to human patients.

Despite increasing sophistication in preclinical modeling, early-phase clinical trials remain indispensable for establishing the true translational potential of nanomedicine in LM-OS. However, the current clinical landscape presents formidable obstacles. LM-OS is a rare disease with limited patient populations, heterogeneous treatment histories, and variable disease burden at diagnosis, complicating patient stratification and trial enrollment. Moreover, conventional endpoints such as overall survival or radiographic response may not adequately capture the benefits of nanomedicine approaches that aim to delay metastatic progression, modulate immunity, or improve quality of life. Regulatory considerations, including long-term safety, batch reproducibility, and combination with standard-of-care therapies, further add to trial complexity.

Bridging the translational gap will therefore require tighter integration of biologically informed nanocarrier design with predictive preclinical models and clinically realistic trial strategies. Approaches such as harmonizing murine and canine data, incorporating pharmacodynamic biomarkers, and designing adaptive early-phase trials tailored to metastatic disease may be essential for converting preclinical promise into meaningful clinical benefit for patients with LM-OS.

## 6 Perspectives

**Fig. 9:**
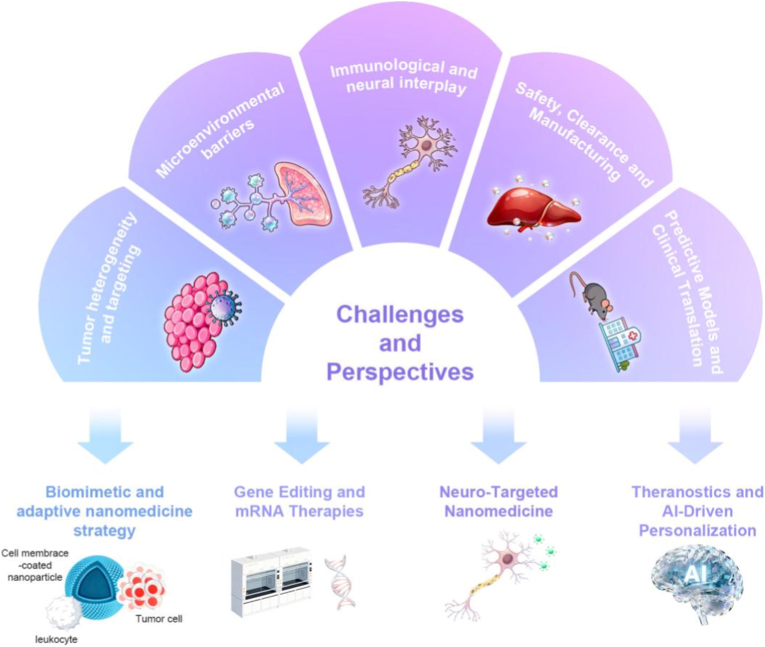
Challenges and future directions of precision nanomedicine for lung metastatic osteosarcoma. Schematic illustration of key biological and translational barriers in LM-OS, including tumor heterogeneity, microenvironmental constraints, immunological and neural interplay, safety and clearance, and model predictivity, together with emerging nanomedicine strategies aimed at addressing these challenges.

Building upon the challenges outlined above, future advances in nanomedicine for LM-OS are expected to emerge from strategies that integrate biological insight with adaptive engineering and data-driven optimization. As summarized in Fig. 9, several converging directions, biomimetic and adaptive nanomedicine, gene editing and mRNA therapies, neuro-targeted nanomedicine, and theranostics coupled with AI-driven personalization, are poised to reshape the translational landscape. Rather than incremental refinements, these approaches aim to fundamentally realign nanocarrier design with disease complexity and clinical reality.

### 6.1 Biomimetic and adaptive nanomedicine strategy

One of the most promising future directions lies in biomimetic and adaptive nanocarrier platforms, which directly address tumor heterogeneity, immune clearance, and microenvironmental barriers [190]. To effectively counter the spatial and temporal heterogeneity of LM-OS, future developments are likely to move beyond single-source membranes toward hybrid or programmable biomimetic interfaces, in which membranes from multiple cell types are combined or supplemented with synthetic components to fine-tune rigidity, permeability, and ligand presentation. This multi-source hybridization strategy enables the simultaneous targeting of diverse sub-clones and phenotypic variants within the metastatic lesion, providing a broader therapeutic coverage that single-target platforms often fail to achieve. Importantly, next-generation designs may incorporate environment-responsive exposure mechanisms, enabling dynamic presentation of targeting ligands or adhesive domains in response to pH, enzymatic activity, or redox conditions within metastatic niches [191].

Exosome-mimetic nanoparticles represent a related but more controllable platform [192]. Produced via extrusion or microfluidic assembly, these systems retain key targeting and communication features of natural exosomes while offering improved reproducibility and scalability [193]. Looking forward, biomimetic nanomedicine is expected to evolve toward adaptive platforms that actively sense and respond to microenvironmental cues, rather than passively mimicking biological surfaces. Furthermore, realizing the clinical potential of these adaptive platforms necessitates a shift in preclinical evaluation methodologies. Future research models must move beyond conventional murine systems toward more sophisticated platforms, such as humanized patient-derived xenograft models and organ-on-a-chip technologies. These advanced models, particularly when integrated with real-time molecular imaging, can more faithfully simulate the complex human metastatic cascade and the heterogeneous responses of metastatic nodules to therapeutic intervention, thereby providing more robust evidence for translational feasibility.

### 6.2 Gene editing and mRNA-based nanotherapeutics

Gene editing and mRNA-based therapies represent a transformative opportunity for LM-OS, particularly in addressing drug resistance, immune evasion, and metastatic persistence. CRISPR/Cas-based systems [194] offer the potential for permanent disruption of oncogenic drivers or restoration of tumor suppressor pathways, while mRNA therapeutics enable transient, tunable expression of therapeutic proteins such as cytokines, tumor antigens, or immune modulators [195].

Nanocarriers are indispensable for the clinical deployment of these modalities, providing protection from nuclease degradation, facilitating intracellular delivery, and reducing off-target exposure. The clinical success of lipid nanoparticles [196] in mRNA vaccine delivery has established a viable manufacturing and regulatory precedent, accelerating interest in adapting similar platforms for cancer therapy. In osteosarcoma, future targets may include genes involved in angiogenesis, immune suppression, and metastatic signaling, such as VEGFA, PD-L1, STAT3, or components of hypoxia and stress-response pathways [197].

Looking ahead, combinatorial strategies integrating gene editing or mRNA delivery with conventional chemotherapy, immunotherapy, or targeted agents may prove particularly effective in eliminating resistant clones and preventing relapse. Key challenges, including off-target editing, immunogenicity of gene-editing components, and delivery efficiency in metastatic lesions, will require continued innovation in nanocarrier design and dosing strategies.

### 6.3 Neuro-targeted nanomedicine as an emerging opportunity in LM-OS

Neuro-targeted intervention is emerging as a biologically substantiated, yet nanomedicine-underutilized, axis for LM-OS. Recent cancer neuroscience studies have moved beyond correlative observations to provide causal evidence that sensory innervation and neurotrophin signaling actively regulate osteosarcoma progression and pulmonary metastatic burden. In particular, a single-cell and multi-omics framework linked tumor-associated sensory neurons with osteosarcoma vascularization and microenvironmental remodeling, and genetic/pharmacologic perturbation of TrkA signaling significantly reduced tumor-associated innervation, angiogenic signaling (including Calcitonin Gene-Related Peptide (CGRP)–VEGF-related pathways), and metastasis, with a measurable reduction of lung metastatic burden in vivo [198].

Mechanistically, the NGF–Trk axis has also been directly implicated in osteosarcoma migratory and metastatic phenotypes. Clinical and transcriptomic analyses have identified NGF as highly expressed in osteosarcoma and associated with metastasis-related programs, providing a human disease–anchored rationale for therapeutically targeting neurotrophin signaling [199]. Complementary experimental work further supports that NGF can promote osteosarcoma progression and immunosuppressive features (e.g., M2-like macrophage association), and that TRK inhibition (e.g., larotrectinib) can antagonize NGF-driven tumor-promoting effects in vivo, collectively reinforcing the drugability of this neurotrophin axis [200,201]. Taken together, these data establish that neuromodulation, especially along the NGF/TrkA–sensory neuron axis, has transitioned from hypothesis to validated anti-metastatic biology in osteosarcoma, while also exposing a clear translational gap: most demonstrations rely on genetics or systemic pharmacology rather than precision delivery.

Looking forward, nanomedicine development in this area should prioritize: (1) target selection tied to LM-OS biology (e.g., NGF–TrkA signaling nodes; CGRP-related neurovascular mediators), consistent with in vivo metastasis evidence; (2) delivery-route matching (lung-local inhalation for pulmonary nodules; bone-anchored or peri-neural depots for primary lesions); and (3) systems-level combination design, recognizing that neural, immune, and vascular programs are co-regulated, an idea supported by reports linking neurotrophin signaling with immune remodeling in osteosarcoma. Collectively, neuro-targeted nanomedicine should be positioned as a forward-looking, high-upside direction for LM-OS-grounded in a rapidly strengthening mechanistic evidence base, yet still awaiting systematic, delivery-centric optimization to translate neuromodulation into a clinically scalable anti-metastatic strategy.

### 6.4 Theranostics and AI-driven personalization

The convergence of nanomedicine with diagnostics and artificial intelligence is expected to drive a shift toward truly personalized therapy for LM-OS [[202], [203], [204]]. Theranostic nanoparticles incorporating imaging agents, such as iron oxide for MRI, radionuclides for PET, or near-infrared fluorophores, enable real-time tracking of biodistribution, tumor accumulation, and therapeutic response. When coupled with stimuli-responsive drug release, such systems can create adaptive treatment loops in which therapy is dynamically adjusted based on in vivo feedback [205].

Advances in computational modeling and artificial intelligence further expand this paradigm. Machine learning algorithms trained on experimental and clinical datasets can predict relationships between nanoparticle physicochemical properties and biological outcomes, guiding rational optimization of size, shape, surface chemistry, and ligand density. Moreover, AI is poised to revolutionize preclinical research models by bridging the gap between animal data and human clinical outcomes. Future developments will increasingly utilize AI-driven computational modeling to simulate the complex pulmonary vascular environment and the diverse cellular interactions within metastatic nodules. By integrating AI with advanced imaging techniques, researchers can develop more predictive animal models that faithfully reflect the multi-organ crosstalk and the evolution of metastatic heterogeneity.

At the patient level, systems biology approaches incorporating genomic, proteomic, and metabolomic data may identify biomarkers that inform nanocarrier selection and payload design. Integration of AI with high-throughput screening platforms and digital twin technologies may substantially accelerate the discovery of formulations tailored to the specific metastatic burden and the evolving microenvironmental landscape of individual patients. Ultimately, this convergence could enable AI-assisted precision nanomedicine, in which therapeutic platforms are matched to individual tumor profiles, metastatic burden, and microenvironmental features.

Realizing these future directions requires interdisciplinary collaboration among oncologists, materials scientists, immunologists, neuroscientists, engineers and data scientists. Robust regulatory frameworks must accommodate the complexity of nanotherapeutics and provide clear pathways for approval. Partnerships with industry and funding agencies are essential to move promising candidates from bench to bedside.

## 7 Conclusion

Lung metastatic osteosarcoma remains a highly lethal disease driven by profound tumor heterogeneity, microenvironmental barriers, immune suppression, and neural regulation, which collectively limit the effectiveness of conventional systemic therapies. Precision nanomedicine provides a powerful framework to address these challenges by enabling targeted delivery, microenvironment-responsive activation, and integrated modulation of metastatic niches. Accumulating preclinical evidence supports a shift from material-centered formulations toward biology-driven, disease-matched nanocarrier designs. Although substantial translational challenges remain, continued advances in biomimetic platforms, gene-based therapeutics, neuro-targeted strategies, and personalized nanomedicine hold promise for improving clinical outcomes and quality of life in patients with lung metastatic osteosarcoma.

As we move toward translating biology-driven nanodesign into clinical practice, we propose a strategic roadmap defined by two pivotal phases over the next eight years. In the immediate 1-4 years, the translational focus will shift toward Phase I/II clinical evaluation, prioritizing the safety, pharmacokinetics, and initial therapeutic efficacy of nanocarriers specifically engineered to bypass pulmonary biological barriers. These early-stage milestones aim to establish robust dosing regimens and assess immune-modulatory responses in patient cohorts, bridging the gap between sophisticated laboratory prototypes and human physiology. Concurrently, refining patient-stratification biomarkers will be essential to align these biology-driven designs with the heterogeneous landscape of metastatic osteosarcoma.

In the subsequent 4-8 years, the trajectory will advance through Phase III trials and regulatory certification, targeting large-scale validation of overall survival benefits and manufacturing scalability (CMC). This phase represents the critical transition from clinical proof-of-concept to widespread bedside application, necessitating rigorous comparison with current standard-of-care regimens. While this timeline remains ambitious, the synchronized evolution of material science and cancer biology provides a feasible path forward. Ultimately, overcoming the inherent barriers of tumor heterogeneity and immune clearance through collaborative innovation will redefine the therapeutic paradigm, offering renewed hope for long-term remission in patients with LM-OS.

## CRediT authorship contribution statement

**Haozheng Li:** Conceptualization, Formal analysis, Investigation, Writing – original draft, Writing – review & editing. **Jing Luo:** Conceptualization, Investigation, Methodology, Writing – original draft, Writing – review & editing. **Qianli Wang:** Conceptualization, Investigation, Methodology, Writing – original draft, Writing – review & editing. **Yang Zhang:** Investigation, Methodology, Writing – original draft, Writing – review & editing. **Qiujiang Li:** Investigation, Methodology, Writing – review & editing. **Xiaoyong Wang:** Investigation, Methodology, Writing – review & editing. **Gang Liu:** Conceptualization, Funding acquisition, Project administration, Supervision, Writing – review & editing. **Changrong Shi:** Conceptualization, Funding acquisition, Investigation, Project administration, Supervision, Writing – review & editing. **Wei Zhang:** Conceptualization, Funding acquisition, Project administration, Supervision, Writing – review & editing.

## Declaration of competing interest

The authors declare that they have no known competing financial interests or personal relationships that could have appeared to influence the work reported in this paper.

## Data Availability

No data was used for the research described in the article.

## Abbreviations

LM-OS: Lung metastatic osteosarcoma
OS: Osteosarcoma
CCL5: C-C Motif Chemokine Ligand 5
CXCL12/SDF-1: C-X-C Motif Chemokine Ligand 12
RUNX2: Runt-Related Transcription Factor 2
TGF-β: Transforming Growth Factor-beta
IL-6: Interleukin-6
VEGF: Vascular Endothelial Growth Factor
NGF: Nerve Growth Factor
EGFR: Epidermal Growth Factor Receptor
AEG-1: Astrocyte Elevated Gene-1
integrin αvβ3: Integrin alpha-V beta-3
VEGFR: Vascular Endothelial Growth Factor Receptor
CD133: Cluster of Differentiation 133
RANKL: Receptor Activator of Nuclear Factor-κB Ligand
PI3K-AKT: Phosphoinositide 3-Kinase-AKT Serine
MAPK: Mitogen-Activated Protein Kinase
JAK-STAT: Janus Kinase- Signal Transducer and Activator of Transcription
c-Met: MET Proto-Oncogene, Receptor Tyrosine Kinase
CD47: Cluster of Differentiation 47
EGFR-SNPs: EGFR aptamer-functionalized salinomycin-loaded nanoparticles
STP: Sarcoma-Targeting Peptide
CD31: Cluster of Differentiation 31
PAS: Periodic Acid-Schiff
H&E analysis: Hematoxylin and Eosin stain analysis
ROS: Reactive Oxygen Species
GSH: Glutathione
ECM: Extracellular Matrix
LMWH: Low Molecular Weight Heparin
PEG: Polyethylene Glycol
EPR: Enhanced Permeability and Retention effect
PMN: Pre-metastatic Niche
VEGFR2: Vascular Endothelial Growth Factor Receptor 2
sEVs: small Extracellular Vesicles
MMP: Matrix Metalloproteinase
MDSCs: Myeloid-Derived Suppressor Cells
TAMs: Tumor-Associated Macrophages
Tregs: Regulatory T cells
CTL: Cytotoxic T Lymphocyte
ICB: Immune Checkpoint Blockade
PLGA: Poly(lactic-co-glycolic acid)
DFMO: α-difluoromethylornithine
CD206: Cluster of Differentiation 206
STAT3: Signal Transducer and Activator of Transcription 3
ICD: Immunogenic Cell Death
CCL2: C-C Motif Chemokine Ligand 2
IL-13: Interleukin-13
cGAS-STING: Cyclic GMP-AMP Synthase-Stimulator of Interferon Genes
PD-1: Programmed Cell Death Protein 1
PD-L1: Programmed Cell Death-Ligand 1
IL-10: Interleukin-10
PDT: Photodynamic Therapy
PCT: Photocatalytic Therapy
mtROS: mitochondrial Reactive Oxygen Species
NADH: Nicotinamide Adenine Dinucleotide
ATP: Adenosine Triphosphate
HMGB1: High Mobility Group Box 1
IL-12: Interleukin-12
IL-2: Interleukin-2
NK-cell: Natural Killer cell
CGRP: Calcitonin Gene-Related Peptide
TRK: Tropomyosin Receptor Kinase
